# Enviromic Assembly Increases Accuracy and Reduces Costs of the Genomic Prediction for Yield Plasticity in Maize

**DOI:** 10.3389/fpls.2021.717552

**Published:** 2021-10-07

**Authors:** Germano Costa-Neto, Jose Crossa, Roberto Fritsche-Neto

**Affiliations:** ^1^Department of Genetics, “Luiz de Queiroz” Agriculture College, University of São Paulo (ESALQ/USP), Piracicaba, Brazil; ^2^Institute for Genomic Diversity, Cornell University, Ithaca, NY, United States; ^3^Biometrics and Statistics Unit, International Maize and Wheat Improvement Center (CIMMYT), Mexico City, Mexico; ^4^Colegio de Posgraduado, Mexico City, Mexico; ^5^Breeding Analytics and Data Management Unit, International Rice Research Institute (IRRI), Los Baños, Philippines

**Keywords:** genomic selection, adaptability, genotype × environment, climate-smart, selective phenotyping

## Abstract

Quantitative genetics states that phenotypic variation is a consequence of the interaction between genetic and environmental factors. Predictive breeding is based on this statement, and because of this, ways of modeling genetic effects are still evolving. At the same time, the same refinement must be used for processing environmental information. Here, we present an “enviromic assembly approach,” which includes using ecophysiology knowledge in shaping environmental relatedness into whole-genome predictions (GP) for plant breeding (referred to as enviromic-aided genomic prediction, E-GP). We propose that the quality of an environment is defined by the core of environmental typologies and their frequencies, which describe different zones of plant adaptation. From this, we derived markers of environmental similarity cost-effectively. Combined with the traditional additive and non-additive effects, this approach may better represent the putative phenotypic variation observed across diverse growing conditions (i.e., phenotypic plasticity). Then, we designed optimized multi-environment trials coupling genetic algorithms, enviromic assembly, and genomic kinships capable of providing *in-silico* realization of the genotype-environment combinations that must be phenotyped in the field. As proof of concept, we highlighted two E-GP applications: (1) managing the lack of phenotypic information in training accurate GP models across diverse environments and (2) guiding an early screening for yield plasticity exerting optimized phenotyping efforts. Our approach was tested using two tropical maize sets, two types of enviromics assembly, six experimental network sizes, and two types of optimized training set across environments. We observed that E-GP outperforms benchmark GP in all scenarios, especially when considering smaller training sets. The representativeness of genotype-environment combinations is more critical than the size of multi-environment trials (METs). The conventional genomic best-unbiased prediction (GBLUP) is inefficient in predicting the quality of a yet-to-be-seen environment, while enviromic assembly enabled it by increasing the accuracy of yield plasticity predictions. Furthermore, we discussed theoretical backgrounds underlying how intrinsic envirotype-phenotype covariances within the phenotypic records can impact the accuracy of GP. The E-GP is an efficient approach to better use environmental databases to deliver climate-smart solutions, reduce field costs, and anticipate future scenarios.

## Introduction

Environmental changing scenarios are a challenge for agricultural research. Developing climate-smart solutions in a time-reduced and cost-effective manner is crucial to minimize economic and environmental impacts in farm fields (Tigchelaar et al., 2018; Cortés et al., 2020; Ramirez-Villegas et al., 2020). All these strategies must be linked with the characterization of growing conditions of crops (Xu, 2016) because it allows for a deeper understanding of how the environmental signal is a driver to shape the past, present, and future phenotypic variations observed in farm fields (e.g., Cooper et al., 2014; Ramirez-Villegas et al., 2018; Heinemann et al., 2019; de los Campos et al., 2020; Antolin et al., 2021; Costa-Neto et al., 2021b). In plant breeding research, mostly based on the selection of best-evaluated genotypes in a certain experimental network, this approach discriminates which genetic and non-genetic factors affect adaptative responses and yield performance. Thus, a conscious and well-conducted environmental characterization is crucial to bridge the results obtained in some experimental networks and expectations for the target population of environments (TPEs) of the breeding program (e.g., Chenu et al., 2011; Heinemann et al., 2015, 2019; Crespo-Herrera et al., 2021).

The not so fresh, yet underused, field of “envirotyping” (environmental + typing, Cooper et al., 2014; Xu, 2016) emerges to not only deliver reliable data for purposes of environmental characterization but also for enriching the breeding analytics routines (Costa-Neto and Fritsche-Neto, 2021) and closing the gap between breeding goals and agronomic development (Cooper and Messina, 2021). Consequently, new ways to establish a biologically accurate approach for predicting a given growing environment, as well as its relationship with TPE major conditions, have been better understood by quantifying the impact and frequency of the major environment-types (envirotypes) across years or locations (e.g., Chenu et al., 2011; Heinemann et al., 2019; Antolin et al., 2021; Cooper et al., 2021). Furthermore, this might also lead to a better understanding of the quality of a certain environment (e.g., a field trial) in providing representative phenotypic records to support selection purposes or as a training population set in predictive breeding approaches. The end result is twofold beneficial, both for capitalizing the effects of genotype by environment interaction in targeting cultivars, yet for providing a better comprehension of the environmental drivers acting on the yield plasticity observed on the field trials (Costa-Neto et al., 2021a; Crossa et al., 2021).

Prediction-based tools have leveraged agronomic and modern plant breeding research in the last decade (see Heinemann et al., 2019; Herzmann et al., 2020; Cooper and Messina, 2021 in this edition). Perhaps one of the major contributions of the predictive tools is the better use of good quality phenotypic records for feeding *in silico* platforms, aimed at screening a large number of genotypes and candidate cultivars (Crossa et al., 2017; Messina et al., 2018; Rogers et al., 2021). Whole-genome prediction (GP, Meuwissen et al., 2001) is the most extensively used predictive tool that is already developed and validated for several crop species and application scenarios (e.g., Lorenzana and Bernardo, 2009; Windhausen et al., 2012; Crossa et al., 2017; Morais Júnior et al., 2018; Fonseca et al., 2021). In crops such as maize, its uses have been consolidated to support diverse stages of breeding programs, from the selection of individuals among breeding populations to advanced stages aimed at predicting the performance of single crosses across multiple environments (e.g., Dias et al., 2018; Messina et al., 2018; Alves et al., 2019; Costa-Neto et al., 2021a; Rogers et al., 2021).

Genome prediction platforms in plant breeding were conceived to model genotype-to-phenotype relationships (G-to-P) under specific environmental conditions, such as certain planting dates and standardized management where the genotypes are mated and evaluated at nursery (e.g., Lorenzana and Bernardo, 2009; Windhausen et al., 2012). Thus, it is reasonable to assume that the realized G-to-P relationship might capture a large part of the observed phenotypic variation, although this is environmental-specific, which generates a noisy marker × environment interaction (Burgueño et al., 2012) when we aim to predict multiple growing conditions. Thus, there is an environmental-phenotype covariance intrinsic on each phenotypic records. Consequently, it generates the well-reported lack of accuracy under genotype × environment interaction (G × E) conditions (Crossa et al., 2017). Therefore, novel ways that include environmental data (Heslot et al., 2014; Jarquín et al., 2014; Ly et al., 2018; Gillberg et al., 2019; Millet et al., 2019; Monteverde et al., 2019; Costa-Neto et al., 2021a) and process-based crop growth models (CGMs) (Messina et al., 2018; Robert et al., 2020; Toda et al., 2020; Cooper et al., 2021) are considered the best pathways to fix it in the context of the multi-environmental GP. Most of the success of such approaches lies in understanding the ecophysiology interplay between genomics diversity and environment variation (Gage et al., 2017; Li et al., 2018; Guo et al., 2020; Costa-Neto et al., 2021b).

In addition to possible accuracy gains, the explicit integration of enviromic and genomic sources is also an easy way to lead GP to a wide range of novel dimensions of applications (Crossa et al., 2021), such as envisaging the performance of crops under untested growing conditions (de los Campos et al., 2020; Guo et al., 2020; Jarquin et al., 2020; Costa-Neto et al., 2021a), optimizing MET networks (Rincent et al., 2017a,b) and screening genotype-specific reaction-norms for key environmental factors (Ly et al., 2018; Millet et al., 2019). This is an excellent advance for predictive breeding (i.e., the range of prediction-based selection tools for crop improvement) because it reduces the time and cost of the research pipelines while better supporting the selection of adapted genotypes for target scenarios. However, to the best of our knowledge, most of the current studies on this topic vary in accuracy and applicability, mostly because of three aspects:(1) processing protocols used to translate raw data into explicit environmental covariables (ECs) with biological meaning for explaining G × E over complex traits, (2) the lack of a widely-used envirotyping pipeline that not only supports the design of field trials but also increases the accuracy of trained GP models, and in addition, (3) for most biological-enriched predictive platforms, such as those enabled by CGMs, there is a possible limitation due to increased demand for the phenotyping of additional intermediate phenotypes (i.e., biomass accumulation and partitioning, specific leaf area), which can involve managed iso-environments and expert knowledge on crop modeling (Cooper et al., 2016; Robert et al., 2020; Toda et al., 2020). The latter can be expensive or difficult for plant research programs in developing countries, with low budgets to increase the phenotyping network and install environmental sensors. In addition, most developing countries are located in regions where environments are subject to a broader range of mixed stress factors, such as drought and heat stresses in combination with nutrient limitation conditions.

Therefore, in this study, we revisit Shelford's Law (Shelford, 1931) and other ecophysiology concepts that can provide foundations for translating raw environmental information into an enviromic source for predictive breeding, hereafter denominated *enviromic assembly*. The benefits of using the so-called “enviromics-aided GBLUP” (E-GP) under existing experimental networks are then presented, followed by a proof-of-concept application of E-GP for optimizing field-based phenotyping. Finally, we benchmark E-GP with the traditional genomic-best unbiased prediction (GBLUP) to discuss the benefits of enviromic data to reproduce the expected G × E pattern, which seems to provide a cost-effective platform to screen the yield plasticity of genotypes.

## Materials and Methods

The Materials and Methods section is organized in the following manner: First, we briefly address the concepts underlying the novel approach of *enviromic assembly* inspired by Shelford's Law. Then, we describe the data sets, along with the statistical models and prediction scenarios used, to show the benefits of enviromics in GP across multi-environment trials (METs). Finally, we present a scheme to optimize phenotyping efforts in training GP over MET and support the screening for the yield plasticity of maize single crosses.

### Theory: Adapting the Shelford Law of Minimum

**Figure 1:**
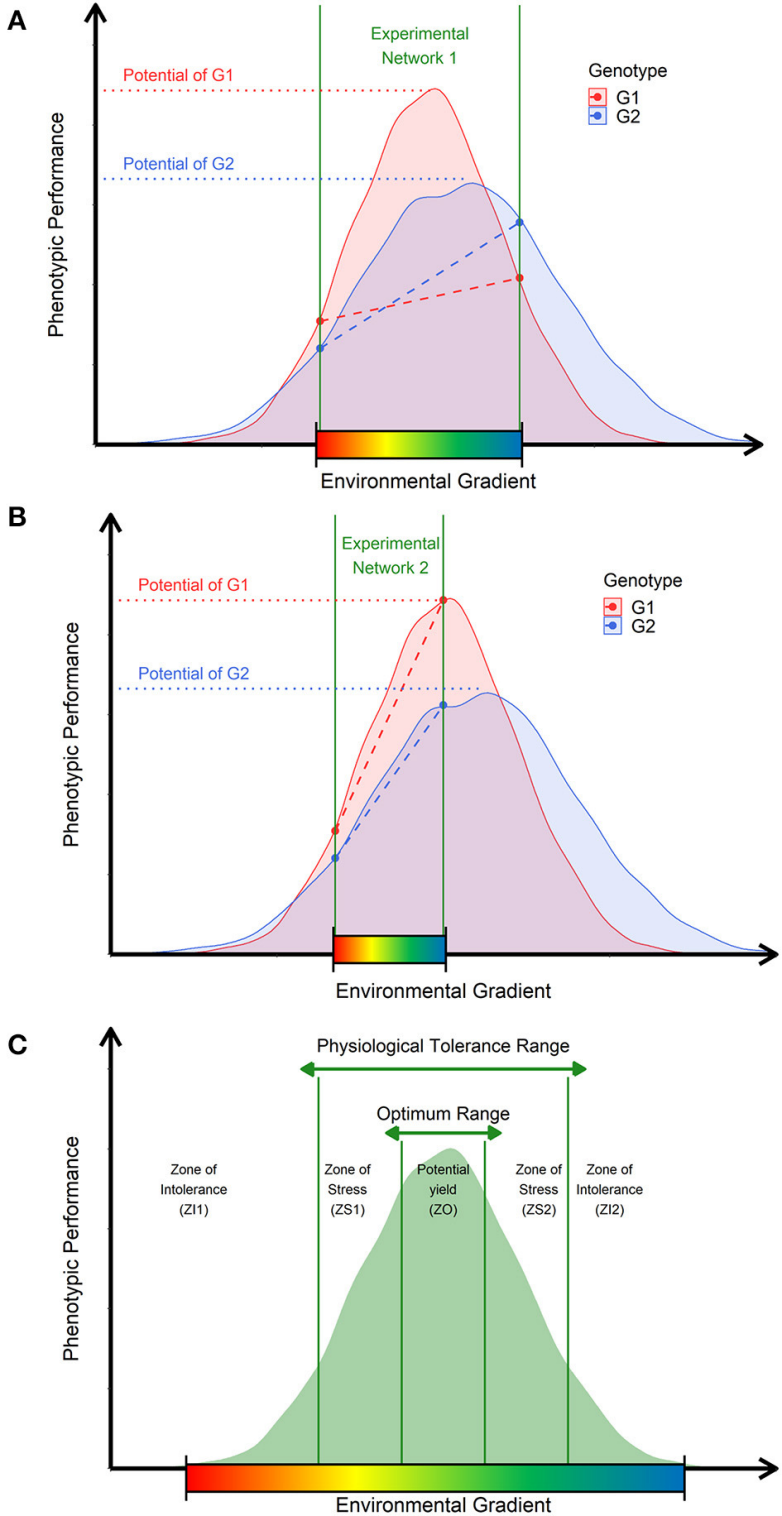
Ecophysiological insights to translate raw environmental data into enviromic sources. **(A)** Representation of an experimental network involving an unknown number of environments from a theoretical target population of environments (TPEs) and two genotypes (G1 and G2). The range of the environmental gradient is delimited by the space between the two vertical green lines. Each genotype has a non-linear function describing the genetic limits of phenotypic plasticity (curves) and genetic potential (horizontal dotted lines) of a given trait. Diagonal dotted lines denote the observed reaction norm experienced by the genotypes. **(B)** Representation of a second experimental network involving the same genotypes, but different environments were sampled from the theoretical TPE. **(C)** Adaptation of Shelford's Law of Tolerance, describing the cardinal (or biological) genetic limits (vertical green lines) to determine the amount of the factor that results in different adaptation zones. Across these zones, crop performance is described by zones of stress caused by deficit or excess (physiological tolerance range) and zones of optimal growing conditions that allow the plants to express the genetic potential for a given trait (optimum range). The core of possible environmental variations is contemplated as putative phenotypic plasticity for a given genotype, germplasm, or crop species.

As an exemplification, please consider two experimental networks (MET) of the same target population of environments (TPEs, e.g., different locations, years, and crop management) covering two distinct ranges of environmental factors (colorful gradient bar) (Figure 1). Then, consider two distinct genotypes evaluated under both METs (G1, G2), in which their putative response curves of phenotypic plasticity (Allard and Bradshaw, 1964) can also be expressed as different linear reaction-norms (dotted lines), which consequently results in distinct observable G × E patterns across METs (Figures 1A,B). For MET 1 (Figure 1A), both genotypes are experiencing a wider range of possible growing conditions (large interval between the two vertical solid lines), which results, in this case, in a possible crossover G × E pattern. Conversely, in MET 2 (Figure 1B), the range of growing conditions is different. Thus, it is expected that the same genotypes will also produce a distinct G × E pattern, which is, in this case, a non-crossover. Therefore, it is feasible to conclude that, although the genetic variation is essential for modeling the potential phenotypic plasticity of genotypes (curves, Figures 1A–C), the diversity of environmental growing conditions dictates the observable G × E patterns (Bradshaw, 1965). Thus, by bringing these observations into the GP context, we envisage that most decisions guided by MET GP models might be unbiased with the quality and diversity of growing conditions are not well-accounted in the modeling approach.

Currently, approaches such as CGM aim to mechanistically reproduce phenotypic plasticity curves in a non-linear way. Conversely, benchmark reaction norm models try to reproduce the observable reaction norm in a linear way. Both approaches can achieve adequate results (e.g., Cooper et al., 2016; Ly et al., 2018; Heinemann et al., 2019; Millet et al., 2019; Monteverde et al., 2019; Jarquin et al., 2020; Toda et al., 2020; Antolin et al., 2021), although, to the best of our knowledge, we have observed three key issues: (1) the quality of the linear modeling of a reaction norm depends on the diversity of METs, and thus, on the range of environmental conditions evaluated, which consequently implicates that the screened impact of environmental factors is MET-specific (not TPE-specific) and varies across years; (2) A CGM demands greater phenotyping effort for training genotype-specific parameters capable of reproducing the *achievable* phenotypic plasticity, from a reduced core of phenotypic records collected from field trials in near-iso environments (e.g., well-watered conditions vs. water-limited conditions for same planting date and management), which, for some regions or crops, can limit the applicability of the method, even if it is a biologically accurate way to reproduce yield plasticity for certain scenarios such as drought stress; (3) the use of reaction norm models trained from high technological and well-designed phenotyping platforms might be efficient to collect reliable environmental phenotype associations, but it might not be feasible for certain regions of the world with limited resources to invest in precision (and expensive) phenotyping efforts. Because of this, there is a need to develop a cheap and easy way to approach environmental diversity, translating it into a source of data capable of mimicking the impact of environmental range in the expression of phenotypic plasticity in the current GP platforms.

We understand that Shelford's Law of Tolerance (Shelford, 1931) is suitable for explaining how the environmental signals are a drive source of the phenotypic plasticity in plants. It can inspire the implementation of a cost-effectively pipeline for processing raw environmental data (Figure 1C). It states that the adaptation of a target population (e.g., germplasm) is modulated as a certain range of minimum, maximum, and optimum threshold limits achieved over an environment gradient (vertical solid green lines). Thus, the potential phenotypic plasticity (curves) of genotypes is not regarded as a linearized reaction norm variation across an environmental gradient (Arnold et al., 2019); instead, it is a non-linear curve of variation, which can be summarized into a discrete distribution, based on the cardinal thresholds for each biophysical factor, with well-documented ecophysiological relevance. Therefore, crops may experience stressful conditions because of the excess or lack of a certain environmental factor (e.g., temperature, water), which depends on cardinal thresholds (vertical solid green lines in Figure 1C) for each plant species, germplasm, genetic pool, and even varies according to the lifetime of a crop, development stages. Consequently, the expected variation under environmental conditions across different field trials can be visualized as a core of environment types (envirotypes) acting consistently yet varying in impact and due to genetic-specific sensibility, as preconized in CGMs. Finally, the quality of a certain growing condition depends on the balance between crop necessity and resource availability, which can be modeled as a *quantity of resources* and its *frequency* across time and space. This came with the idea of separating the environmental inputs as *constant effects*, such as the type of treatments in a trial (e.g., fertilizer inputs) and *transitory effects* variables, such as weather events (e.g., heat stress).

From these concepts, we observe that by envirotyping (e.g., typing the profiles of a particular environment) we can visualize the contribution of the observable G × E pattern as an end-result of the shared frequency of envirotypes across different field trials. Thus, the *envirome* of a certain experimental network or TPE (the core of possible growing conditions) can be mathematically assembled in three steps: (1) collecting large-scale environmental data, (2) processing this raw data in envirotyping entries for each real or virtual environment, and (3) processing the envirotyping-derived entries to achieve theoretical relatedness between the buildup of different environments from the shared frequency of envirotypes. Thus, the expected envirotypes can be designed relying on the adaptation zones inspired by the model proposed here, based on Shelford's Law, in which we can envisage the process of deriving environmental covariables for GP in an ecophysiological-smart way.

### Proof-of-Concept Data Sets

Grain yield data (Mg per ha) collected from two distinct sets of maize hybrids (single crosses of inbred lines) were used as a proof of concept. The data sets were originated in Brazil from different germplasm sources developed under tropical conditions (hereafter referred to as Multi-Regional and N-levels). The experimental design, cultivation practices, and fundamental statistical analysis are given in Bandeira e Souza et al. (2017) and Alves et al. (2019). Below, we provide a short description of the number of genotypes and environments tested and the nature of the genotyping data of this study.

#### Multiregional Set (Five Locations in Different Regions)

The so-called “Multi-Regional set” is based on germplasm developed by the Helix Seeds Company (HEL) in South America. It includes 247 maize lines evaluated in 2015 at five locations in three regions of Brazil (Supplementary Table 1). Genotypic data were obtained using the Axiom Maize Genotyping Array (Affymetrix, Sta. Clara, CA, United States) containing 616 K single-nucleotide polymorphisms (SNPs) (Unterseer et al., 2014). Only SNPs with minor allele frequency >0.05 were considered. Finally, a total of 52, 811 high-quality SNPs that achieved the quality control level were used in further analyses.

#### N-Level Set (Fertilization Levels Across Years and Locations)

The so-called “N-level set” is based on the germplasm developed by the Luiz de Queiroz College of Agriculture of the University of São Paulo (USP), Brazil. A total of 570 tropical maize hybrids were evaluated across eight environments, involving an arrangement of two locations, 2 years, and two nitrogen levels (Supplementary Table 2). The sites of this study involved two distinct edaphoclimatic patterns, i.e., Piracicaba (Atlantic Forest, clay soil) and Anhumas (savannah, silt–sandy soil). Two contrasting nitrogen (N) fertilization levels were managed at each site. One experiment was conducted under ideal N conditions and received 30 kg ha^−1^ at sowing, along with 70 kg ha^−1^ in a coverage application at the V8 plant stage, which is the main recommendation for fertilization in tropical maize growing environments in Brazil. In contrast, the second experiment under low N conditions received only 30 kg ha^−1^ of N at sowing, resulting in N-limited growing conditions. The genotyping data of this set were obtained using the Axiom Maize Genotyping Array (Affymetrix, Sta. Clara, CA, United States) containing 616 K SNPs (Unterseer et al., 2014) and minor allele frequency >0.05. At the end of this process, a total of 54,113 SNPs were considered and used in the further analysis.

### Envirotyping Pipeline

In this section, we present the methods used for data collection, processing, and implementing what we call *enviromic assembly*. This envirotyping pipeline was developed using the functions of the R package EnvRtype (Costa-Neto et al., 2021b) and is available at no cost.

#### Environmental Sensing (Data Collection)

This study used environmental information for the main abiotic plant-environment interactions related to daily weather, soil type, and crop management (available only for the N-level set). Daily weather information was collected from NASA POWER (Sparks, 2018) and consisted of eight variables: rainfall (P, mm day^−1^), maximum air temperature (TMAX, °C day^−1^), minimum air temperature (TMIN, °C day^−1^), average air temperature (TAVG, °C day^−1^), dew point temperature (TDEW, °C day^−1^), global solar radiation (SRAD, MJ per m^2^), wind speed at 2 m (WS, m s^−1^day^−1^), and relative air humidity (RH, % day^−1^). In addition, elevation above sea level was obtained from the Shuttle Radar Topography Mission (SRTM) of NASA. Both sources were imported into R statistical-computational environments using the functions and libraries organized within the EnvRtype package (Costa-Neto et al., 2021b). A third GIS database was used to import soil types from Brazilian soil classification provided by *Empresa Brasileira de Pesquisa Agropecuária* (EMBRAPA), available at http://www.dpi.inpe.br/Ambdata/mapa_solos.php and at the Git Hub tutorial https://github.com/gcostaneto/EGP.

#### Data Processing

Quality control was adopted by removing variables outside the mean ± three SDs and repeated columns. After checking for outliers, the daily weather variables were used to model ecophysiological interactions related to soil-plant-atmosphere dynamics. The thermal-radiation interactions computed potential atmospheric evapotranspiration (ET0) following the Priestley–Taylor method (Priestley and Taylor, 1972). The slope of the curve of saturation vapor pressure (SVP) and vapor pressure deficit (VPD) was computed as given in the food and agriculture organization (FAO) manual (Allen et al., 1998). An FAO-based generic function was used to estimate crop development as a function of days after emergence (DAE). We assume a three-segment leaf growing function to estimate the crop canopy coefficient (Kc) of evapotranspiration using the following Kc values: Kc_1_ (0.3), Kc_2_ (1.2), Kc_3_ (0.35), equivalent to the water demand of tropical maize for initial phases, reproduction phases, and end-season stages, respectively. Using the same three-segment function, we estimate the crop canopy using a leaf area index (LAI) of LAI = 0.7 (initial vegetative phases), LAI = 3 (maximum LAI for tropical maize growing conditions observed in our fields), and LAI = 2 (LAI tasseling stage). We computed the daily crop evapotranspiration (ETc) estimated by the product between ET0 and the Kc from the two estimations. Then, we computed the difference between daily precipitation and crop evapotranspiration as P-ETc.

The apparent photosynthetic radiation intercepted by the canopy (aPAR) was computed following aPAR = SRAD × [1-exp(–k × LAI)], where k is the coefficient of canopy, considered as 0.5 (Sinclair, 2006). Water deficiency was computed using the atmospheric water balance between the input (precipitation) and output of atmospheric demands (crop evapotranspiration). The effect of temperature on radiation use efficiency (F_RUE_) was described by a three-segment function based on cardinal temperatures for maize, using the cardinal temperatures 8°C (Tb_1_, base lower), 30°C (To_1_, base optimum), 37°C (To_2_, upper optimum), and 45°C (Tb_2_, base upper). This function assumes values from 0 to 1, depending on: F_RUE_ = 0 if T_AVG_ ≤ Tb_1_; F_RUE_ = (T_AVG_-Tb_1_)/(To_1_-Tb_1_) if Tb_1_ < T_AVG_ < To_1_; F_RUE_ = 1 if To_1_ < T_AVG_ < To_2_; F_RUE_ = (Tb_2_-T_AVG_)/(Tb_2_-To_2_) if To_2_ < T_AVG_ < Tb_2_; and F_RUE_ = 0 if T_AVG_ > Tb_2_ (Soltani and Sinclair, 2012).

Finally, we sampled each piece of weather and ecophysiological information across five-time intervals in the lifetime of the crop: from emergence to the appearance of the first leaf (V1, 14 DAE), from V1 to the fourth leaf (V4, 35 DAE), from V4 to the tasseling stage (VT, 65 DAE), from VT to the kernel milk stage (R3, 90 DAE), and from R3 to physiological maturity (R6, 120 DAE), in which DAE stands for days after emergence. These time intervals were based on fixed DAE according to our expertise in tropical maize and how its germplasm grows under Brazilian conditions. After emergence to V1, there are two critical vegetative phases in which different absorption rates of soil resources and dry matter production are expected. Radiation and factors of water balance are important for these stages. Then, between V1 and V3, a higher rate of leaf growth is expected, which starts to become faster from V4 until it stops in VT (tasseling stage). A third critical stage begins at the beginning of VT, passing through the milk stage (R1) until R3. At the same time, the sensibility of abiotic stresses, such as heat stress and drought stress, also increases. In this phase, pollination and kernel formation will start, which directly impacts grain yield production. Finally, from R3 to R6, the grains pass the dough (R4) and dent stages (R5), which have less sensibility to most environmental stresses but are still affected by thermal-related factors, which can also accelerate these stages. For adapting the methodology to other crops, we encourage the readers to (1) explore ecophysiology concepts from the literature, especially those related to plant science; (2) incorporate multidisciplinary efforts in agronomic expertise; (3) use crop growth models to establish development stages, if necessary.

#### T Matrix: Envirotype Markers Using Typologies

The raw envirotyping data were used to assemble markers for environmental similarity, depending on the group of ECs. The first group of ECs involves *transitory effect variables*, which vary in the frequency of occurrence, depending on the crop development cycle. Thus, according to the concepts inspired by Shelford‘s Law, we designed the expected envirotypes using the number of inputs required to lead crops in at least three levels of adaptation: (1) stress by deficit, (2) optimum growing conditions, and (3) stress by excess. These levels were defined using cardinal thresholds or frequency tables concerning the growing conditions archived in the range of experimental networks. Then, having reviewed the literature, we considered the intervals for thermal-related variables: 0 to 9°C (death), 9.1 to 23°C (stress by deficit), 23.1 to 32°C (optimum growing conditions), 32.1 to 45°C (stress by excess), and 45 to ∞°C (death). According to our agronomic expertise in the crop, we computed the types of the expected rainfall requirements for tropical maize growing environments: 0 to 10 mm, 10.1 to 20 mm, and 20.1 to ∞ mm. In the same way, for crop evapotranspiration (ETc), we assumed the envirotypes 0–6, 7–10, 10–15, and 16 to ∞ mm.day^−1^. Finally, for F_RUE_, we assumed four impact levels: 0 to 25% (0–0.25), 26 to 50% (0.26–0.5), 51 to 75% (0.51–0.75), and 76 to 100% (0.76–1). Finally, for the remaining variables, we preferred to adopt a simple discretization approach, using a histogram of percentiles (0–25, 26–50, 51–75, and 75–100%) of occurrence for a target envirotype. We understand that, for other crop species or a lack of expertise in the crop or the germplasm, the use of discretization must suffice until we know the genetic thresholds of each limit for each environmental factor better.

Additionally, we also considered the group of *constant effect variables*. This group involved factors related to elevation, crop management, and soil classification in each environment. Soil information was entered as an incidence matrix (0 or 1) based on the occurrence in each environment. In addition, for the N-level set, nitrogen input levels were computed as two discrete classes: ideal, N = 10, and low, N = 30; we entered the same incidence matrix for soil information. Because both sets have a gradient for elevation, we used a histogram of percentiles (0–25, 26–50, 51–75, and 75–100%) as in the transitory group of variables. Finally, each designed envirotype × time interval frequency was used as a qualitative marker of environmental relatedness (hereafter, the T matrix, from *t*ypologies).

#### W Matrix: Assembly Quantitative Covariables

The quantitative descriptors of environmental relatedness are the most common method to include environmental information in GP studies considering reaction norms (e.g., Jarquín et al., 2014; Morais Júnior et al., 2018; Monteverde et al., 2019; Costa-Neto et al., 2021a; Rogers et al., 2021). Jarquín et al. (2014) proposed the creation of the so-called environmental relatedness kinship (**K**_E_), carried out with a matrix of quantitative environmental covariables (the W matrix; thus we refer to this environment kinship as **K**_E,W_). Here, the pattern of similarity in ***K***_E,W_ was captured using percentile values (25; 50, and 75%) in each of the five-time intervals of development, as suggested by Morais Júnior et al. (2018) and expanded by Costa-Neto et al. (2021a). As a result, we found 255 and 307 quantitative descriptors for the multiregional and N-level sets, respectively, at the end of the process. In this study, we used ***K***_E,W_ as a benchmark method to test the effectiveness of the ***K***_E,T_ matrix and the total absence of environmental information (baseline genomic model without environmental information; refer to section Baseline additive-dominant GBLUP).

### Statistical Models

From a baseline additive-dominant multi-environment GBLUP (section Baseline additive-dominant GBLUP), we tested four other models, by including two types of enviromic assembly (T or W) and two structures for G × E effects. More details about each statistical model are provided in the next subsections. All the kernel models were fitted using the BGGE R package (Granato et al., 2018) using 15,000 iterations, with 2,000 used as burn-in and using a thinning of 10. This package was used because of the following aspects: (1) it is an accurate open-source software and (2) it can accommodate many kernels in a computation-efficient way.

#### Baseline Additive-Dominant GBLUP

The baseline model includes a fixed intercept for each environment and random genetic variations due to additive and dominance effects and their interaction with the environment. We will refer to this model as just GBLUP, which was modeled as the main effect plus a genomic-by-environment deviation (the so-called G + GE model), as follows:


(1) $$\begin{array}{l} y=1\mu +Z_{E}\beta +Z_{A}u_{A}+Z_{D}u_{D}+u_{AE}+u_{DE}+\varepsilon \end{array}$$

where $\text{y}={\left[{\text{y}}_{1},\cdots \;,{\text{y}}_{\text{n}}\right]}^{\prime }\;$is the vector of observations collected in each of the *q* environments with hybrids, and **1μ+Z**_*E*_**β** is the general mean and the fixed effect of the environments with incidence matrix **Z**_*E*_. The genetic variation was modeled assuming main additive effects (**u**_*A*_), with **u**_*A*_~$N\left(0,{{\text{J}}_{q}⊗\text{K}}_{A}\sigma_{A}^{2}\right)$, plus a random dominance variation (**u**_*D*_), with **u**_*D*_~$N\left(0,{{\text{J}}_{q}⊗\text{K}}_{D}\sigma_{D}^{2}\right)$, where $\sigma_{A}^{2}$ and $\sigma_{D}^{2}$ are the variance components for additive and dominance deviation effects; **Z**_*A*_ and **Z**_*D*_ are the incidence matrixes for the same effects (absence = 0, presence = 1), **J**_*q*_ is a *q* × *q* matrix of 1s, and **⊗** denotes the Kronecker product. G × E effects are modeled using a block diagonal (BD) matrix of the genomic effects, built using **u**_*AE*_~$N\left(0,{{\text{I}}_{q}⊗\text{K}}_{A}\sigma_{A}^{2}\right)$ and **u**_*DE*_~$N\left(0,{{\text{I}}_{q}⊗\text{K}}_{D}\sigma_{D}^{2}\right)$, in which **I**_*q*_ is a diagonal matrix of the *q* × *q* dimension. Residual deviations (**ε**) were assumed as **ε**~$N\left(0,{\text{I}}_{n}\sigma^{2}\right)$, where *n* is the number of genotype-environment observations. Furthermore, the genotyping data were processed in two matrices of additive and dominance effects (Vitezica et al., 2013), modeled with

**A** = {0 = *aa*; 1 = *Aa*; 2 = *AA*} and
$\text{D}=\left\{-2f_{l}^{2}=aa;2f\left(1-f_{l}\right)=\;Aa;-2{f\left(1-f_{l}\right)}^{2}=AA\;\right\}$,

where *f*_*l*_ is the frequency of the favorable allele*A* at locus *l*. Thus, the genomic-related kinships were estimated as follows:


(2) $$\begin{array}{l} \text{K}=\frac{\text{X}{\text{X}}^{\prime }}{tr\left(\text{X}{\text{X}}^{\prime }\right)/\text{r}} \end{array}$$

where ***K*** is a generic representation of the genomic kinship (**K**_*A*_, **K**_*D*_), **X** is a generic representation of the molecular matrix (**A** or **D**), and **r** denotes the number of rows in the **X** matrix. Equation (2) was also used to shape the environmental relatedness kernels using the **T** or **W** matrix. The linear kernel for **K**_E_ was described by Jarquín et al. (2014), which was several other authors named after “**Ω**.” Thus, here, we only tested the difference between the source of enviromics considered for building it and not the merit of the kernel method, as was done in previous studies (Costa-Neto et al., 2021a).

#### GBLUP With Enviromic Main Effects From T Matrix (E-GP)

From equation (1), we added a main environmental relatedness effect, that is, an enviromic main effect carried out with the T matrix (**u**_*E,T*_), as follows:


(3) $$\begin{array}{l} y=1\mu +Z_{A}u_{A}+Z_{D}u_{D}+u_{AE}+u_{DE}+u_{E,T}+\varepsilon \end{array}$$

with **u**_*E,T*_~$N\left({\text{Z}}_{E}\beta ,{\text{K}}_{E,T}{{⊗\text{J}}_{p}\sigma }_{E,T}^{2}\right)$, where **J**_*q*_ is a *p* × *p* matrix of 1s, and **K**_*E,T*_ is the environmental relatedness created and variance component from the T matrix. If non-enviromic sources are considered, the expected value for environments is given by **Z**_*E*_**β** as the baseline model (Costa-Neto et al., 2021a,b). In this model, the effects of G × E are also modeled as the BD genomic matrix. Thus, we refer to this model as “E-GP (BD).” The kernel of the enviromic assembly (**K**_*E,T*_) was built using the panel of envirotype descriptors (T) in the same way as described in equation (2).

From model (3), we substitute the BD for a reaction norm (RN, Jarquín et al., 2014) based on the Kronecker product between the enviromic and genomic kinships (Martini et al., 2020) for additive (**u**_*AE,T*_**)** and dominance effects (**u**_*DE,T*_):


(4) $$\begin{array}{l} y=1\mu +Z_{A}u_{A}+Z_{D}u_{D}+u_{T}+u_{A,T}+u_{D,T}+\varepsilon \end{array}$$

with **u**_*A,ET*_~$N\left(0,{\text{K}}_{E,T}{{⊗\text{K}}_{A}\sigma }_{AE,T}^{2}\right)$ and **u**_*D,ET*_~$N\left(0,{\text{K}}_{E,T}{{⊗\text{K}}_{D}\sigma }_{DE,T}^{2}\right)$, where $\sigma_{AE,T}^{2}$ and $\sigma_{DE,T}^{2}$ are the variance components for enviromic × additive and enviromic × dominance effects, respectively, performed as reaction norms (Costa-Neto et al., 2021a; Rogers et al., 2021). For brevity, this model will be called “E-GP (RN).”

#### GBLUP With Enviromic Main Effects From the W Matrix (W-GP)

Finally, in models (4) and (5), we replaced the source of enviromic assembly derived from T with the same kernel size derived from W, that is, environmental relatedness with **u**_*E,W*_~$N\left({\text{Z}}_{E}\beta ,{\text{K}}_{E,W}{{⊗\text{J}}_{p}\sigma }_{E,W}^{2}\right)$, thus, creating two other models:


(5) $$\begin{array}{l} y=1\mu +Z_{A}u_{A}+Z_{D}u_{D}+u_{AE}+u_{DE}+u_{E,w}+\varepsilon \end{array}$$

and


(6) $$\begin{array}{l} y=1\mu +Z_{A}u_{A}+Z_{D}u_{D}+u_{E,w}+u_{AE,w}+u_{DE,w}+\varepsilon \end{array}$$

$u_{AE,W}∼N(0,K_{E,W}⊗K_{A}\sigma_{AE,W}^{2})$ and $u_{DE,W}\sim N(0,K_{E,W}⊗K_{D}\sigma_{ED,W}^{2})$, where **K**_*E,W*_ and $\sigma_{E,W}^{2}$ are the resulting kinship and variance components estimated for enviromic assembly from the W matrix, respectively. Thus, for brevity, models (5) and (6) will be referred to as “W-GP (BD)” and “W-GP (RN)” (Jarquín et al., 2014), respectively.

### Study Cases for the E-GP Platform

**Figure 2:**
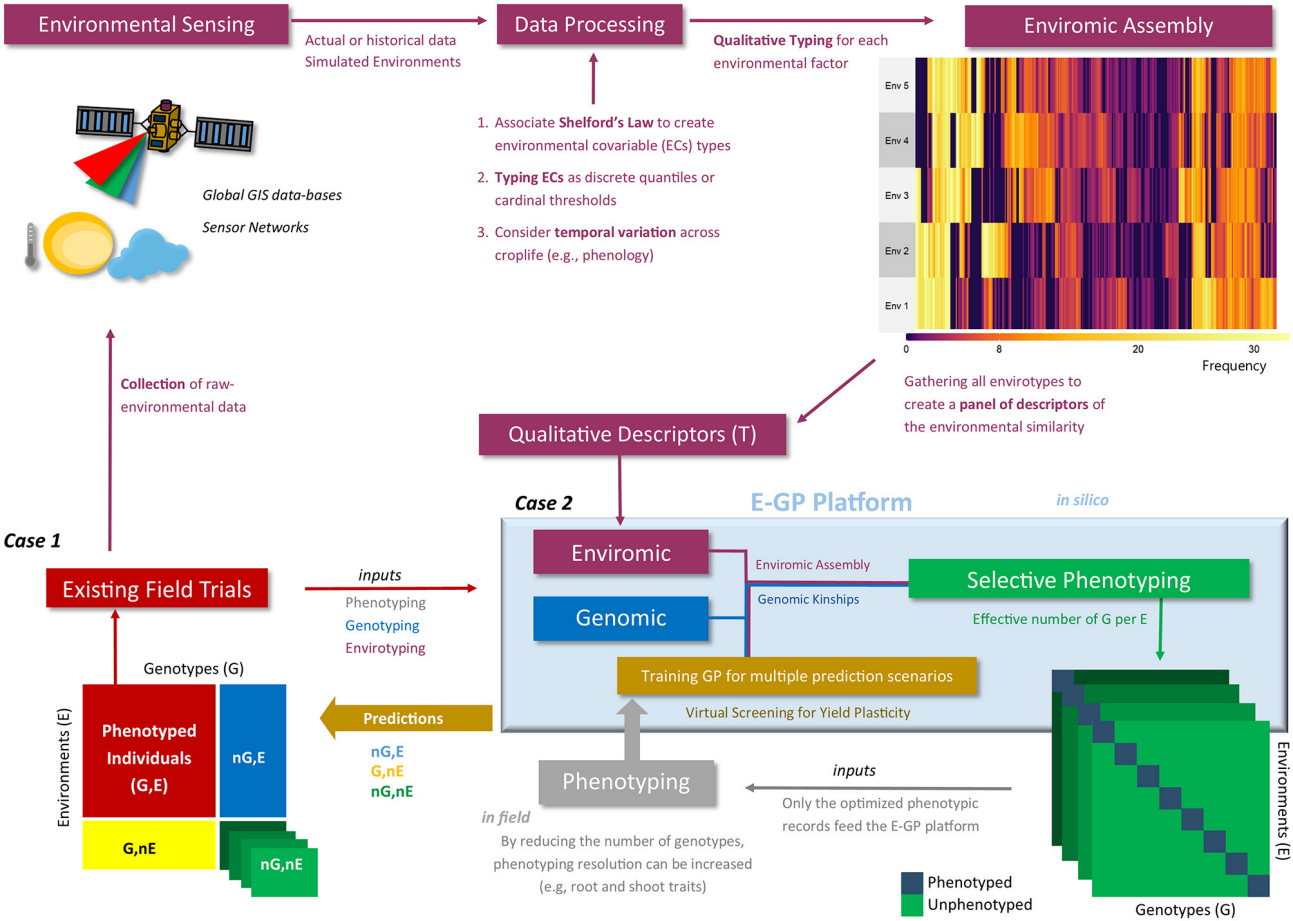
Workflow of the enviromic-aided genomic prediction (E-GP) considering the two study cases (Case 1 and Case 2) of this study. Phenotypic records from existing field trials (red box) are based on observed genotypes (G) in tested growing environments (E). Currently, these data are being used for training prediction models considering untested genotypes at the same conditions (nG, E), especially when we have some type of structure of genetic relationships, such as genomic data (blue colors). In addition, novel growing conditions can be predicted (G, nE and nG, nE) using enviromic sources (wine colors), Case 1. First, raw environmental data are collected from trials involving equipment installed *in situ* (e.g., micro-weather stations) or remote sensing techniques. Then the raw data are processed and translated into an enviromic source that carries some ecophysiology process or statistical distribution of the raw data across time and space. The enviromic assembly is then finalized, in which its product is a matrix of envirotype markers by environments. Taking the T matrix as an example (qualitative descriptors based on typologies), a predictive breeding tool merging genomic, enviromics, and phenotypic data can be trained and deliver predictions for several scenarios of G × E. However, there is a second way to create an E-GP platform, the hereafter Case 2, in which the previously collected genomic and enviromic sources for a given TPE are used to develop *in silico* realizations of the expected G × E for a certain experimental network. Then, optimization algorithms are used to design a selective phenotyping strategy (green box) in which only the most representative genotype-environment combinations are phenotyped and considered for training the E-GP models (gray box). Finally, diverse G × E can also be predicted.

In this study, we conceived two cases to check the possible benefits of involving E-GP in current prediction-based platforms for hybrid testing in maize breeding (Figure 2). The first case (Case 1) consists of the prediction of single-crosses considering diverse sizes of the experimental network in terms of the number of environments considered for the training set. For this case, we dissected the predictive ability over four G × E prediction scenarios. In the second case (Case 2), we envisaged the design of a super-optimized experimental network using the most representative combination of genotypes and environments selected using genomics, enviromic assembly, and genetic algorithms. Then, we envisaged how a small training set (and reduced phenotyping effort) for E-GP and GBLUP might be useful to reproduce the adaptability of maize hybrids for the full-rank MET. Below, we describe in detail each case we studied.

#### Case 1: Expanding the Existing Field Trials

In the first case (Case 1), we adapted a cross-validation scheme to split the global available phenotypic information (*n*), from *p* genotypes and *q* environments, into different training setups. Consequently, four G × E prediction scenarios were created based on the simultaneous sampling of the phenotypic information for *S* genotypes and *R* environments (adapted from Millet et al., 2019). The description and size of each training set are given below:

- G, E refers to predictions of tested genotypes within the experimental network (known genotypes in known environmental conditions). The size of this set is $n_{\left[G,E\right]}=n\times \left(\frac{S}{p}\right)\times \left(\frac{R}{q}\;\right)$;
- *n*G, E refers to predictions of untested (new) genotypes within the experimental network (known environmental conditions). The size of this set is $n_{\left[nG,E\right]}=n\times \left(1-\frac{S}{p}\right)\times \left(\frac{R}{q}\;\right)$;
- G, *n*E, in this scenario, predictions are made under environmental conditions external to those found within the experimental network. However, there is phenotypic information available within the experimental network. The size of this set is $n_{\left[G,nE\right]}=n\times \left(\frac{S}{p}\right)\times \left(1-\frac{R}{q}\;\right);$
- *n*G, *n*E refers to predicting untested(new) genotypes and untested (new) environmental conditions. The size of this size is $n_{\left[nG,nE\right]}=n\times \left(1-\frac{S}{p}\right)\times \left(1-\frac{R}{q}\;\right)$.

Theoretically, if *R*/*q* = 1, then *n*_[*G, nE*]_ = *n*_[*nG, nE*]_ = 0, equal to the commonly used CV1 scheme (prediction of novel genotypes in known environments). Different intensities of *R*/*q* can be sampled, for instance, which permits the testing of setups of experimental networks. Here, we tested three setups for each tropical maize data set. For the N-level set, we made 3/8, 5/8, and 7/8; for the Multi-local set, we made 2/5, 3/5, and 4/5. We assumed the same level of genotype sampling as the training set for all the experimental setups, equal to a fraction of $\frac{S}{p}=\;0.7$. Each training setup was randomly sampled 50 times in order to compute the statistics of prediction quality. For this purpose, two statistics were used to assess the performance of the statistical models over the training setups. First, we calculated Pearson's moment correlation (*r*) between observed (*y*) and predicted (ŷ) values and used the average value for each model and training setup as a predictive ability statistic. Second, to check the ability of GP to replace field trials, we computed the coincidence (CS, in %) between the field-based selection and the selection-based selection of the top 5% best-performing hybrids in each environment.

#### Case 2: Designing Super-Optimized Field Trials

The design of “super-optimized field trials” was based on three steps. First, we computed a full-entry G × E kernel based on the Kronecker product (⊗) between the kernels derived from enviromic assembly-based (**K**_*E,T*_, *q* × *q* environments) and genomic kinship (**K**_*G*_, *p* × *p* genotypes); thus, **K**_*GE,T*_ = **K**_*E,T*_ ⊗ **K**_*G*_, with an *n* × *n* dimension, in which *n* = *pq*. Here, we adopt the additive effects (**K**_*G*_ = **K**_*A*_) as the genomic kinship, despite the benefits of dominance effects in the modeling of G × E. We chose to use only **K**_*A*_ for simplicity, since additive effects seem to be a major genomic-related driver of G × E for grain yield in tropical maize (Dias et al., 2018; Alves et al., 2019; Costa-Neto et al., 2021a; Rogers et al., 2021), a fact that was also observed for Case 1 (see section Case 1: accuracy in predicting diverse G × E scenarios). Later, we applied a single-value decomposition in **K**_*GE,T*_, following ${\text{K}}_{GE,T}=\text{U}\text{V}{\text{U}}^{\text{T}}$, where ***U*** is the total of eigenvalues, and ***V*** is the respective eigenvectors. The number of eigenvalues that explains 98% of the variance present in **K**_*GE,T*_ indicates the number of effective SNPs by envirotype-marker interactions (adapted from Misztal, 2016), which is also the minimum core of genotype-environment combinations (*N*_*GE*_). Thus, the reduced phenotypic information of some genotypes in some environments (*N*_*GE*_) was used to predict a virtual experimental network (*N*_*test*_), involving all remaining single crosses in all the available environments, as given by *N*_*test*_ = *n* − *N*_*GE*_.

Following this step, a genetic algorithm scheme using the design criteria PEV_MEAN_ was used to identify the *N*_*GE*_ entries within the **K**_*GE,T*_. Optimization was implemented using the SPTGA R package (Akdemir and Isidro-Sánchez, 2019) using 100 iterations: five solutions selected as elite parents were used to generate the next set of solutions and mutations of 80% for each solution generated.

### Virtual Screening for Yield Plasticity

Finally, we checked the potentialities of using E-GP to predict the environmental quality and adaptability of each genotype across the environments using only the *N*_*GE*_ phenotypic information. First, the prediction ability was computed for genotypes by correlating the predicted and observed grain yield values across the environments (Costa-Neto et al., 2021a). The second measure was based on the regression slope of the Finlay–Wilkinson adaptability model (Finlay and Wilkinson, 1963). The values of GP were regressed to the observed environmental deviations as follows:


(7) $$\begin{array}{l} M_{ij}={\bar{y}}_{i.}+b_{i}I_{j}+\varepsilon_{ij} \end{array}$$

where *M*_*ij*_ is the expected GP-based mean value of grain yield for the *i*^th^ genotype in the *j*^th^ environment$;\;{\bar{y}}_{i.}$ is the mean genotypic value for the *i*^th^ genotype, *b*_*i*_ is the genotype plastic response across the mean-centered standardized environmental score (*I*_*j*_), and ε_*ij*_ is the variety of residual deviation sources not accounted for in the model. After this step, the Pearson's product-moment correlation between GP-based (${\hat{b}}_{i}$) and phenotypic-enabled estimates were computed as an indicator of the ability to reproduce plastic responses *in silico* for the *p* genotypes. For this, mean squared error is also calculated as:


$$\begin{array}{l} MSE=\overset{p}{\underset{i=1}{\sum }}\frac{{\left(b_{i}-{\hat{b}}_{i}\right)}^{2}}{p} \end{array}$$

All the statistics were computed using the entire data sets, and only the top 5% of genotypes were selected for each environment. The latter aimed to check the efficiency of the E-GP method to produce high-quality virtual screenings for plasticity.

### Data and Code Availability

All the data sets and codes (in R), with a toy example of use, are freely available at https://github.com/gcostaneto/EGP.

## Results

### Case 1: Predicting Diverse G × E Scenarios

**Figure 3:**
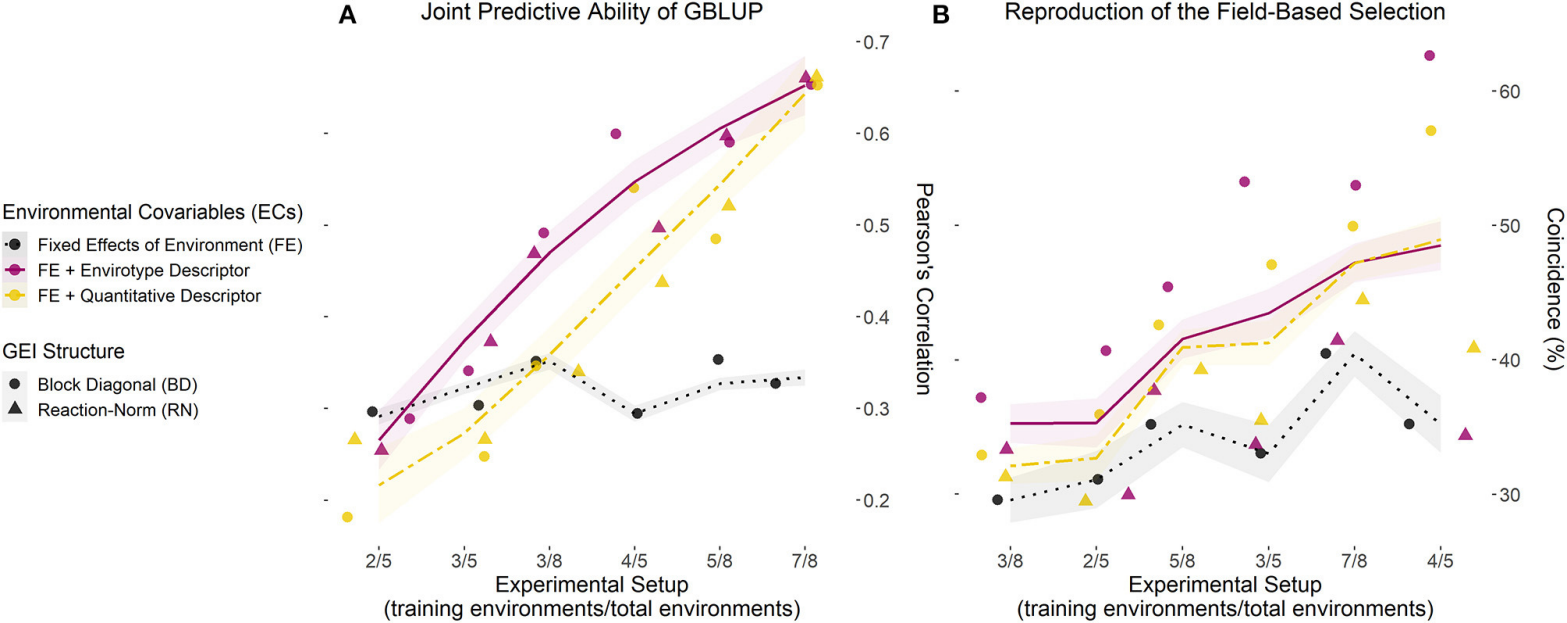
Joint accuracy trends of GP models for each training setup of existing experimental networks. **(A)** Predictive ability computed with the correlation (*r*) between observed (*y*) and predicted (ŷ) values for the grain yield of each genotype in each environment, over three experimental setups (number of environments used/total of environments) for both maize sets (N-level and multilocal), using 70% of the genotypes as a training set and the remaining 30% as a testing set. **(B)** Coincidence index (CS) between the field-based and prediction-based selection of the best 5% genotypes in each environment for the same experimental setups and data sets. Dots and triangles represent the point estimates of predictive ability and CS for models involving a block diagonal genomic matrix for G × E effects (dotted) and an enviromic × genomic reaction norm G × E effect (triangle). Trend lines were plotted from the partial values of each sample (from 1 to 50) and three prediction scenarios (*n*G, E; G, *n*E; and *n*G, *n*E) using the gam () integrated with smoothness estimation in R. Black dotted lines represent the benchmark GBLUP method, considering the effect of the environment as a fixed intercept. Yellow two-dash lines represent the GBLUP involving the main effect from quantitative descriptors (W matrix). Finally, solid dark pink lines represent the GBLUP involving the main effect of envirotype descriptors (T matrix). Thus, the latter represents the E-GP based approach for Case 1 (predictions under existing experimental networks).

The first case tests the effect of experimental setups in providing reliable phenotypic information as training population sets. For this, sample genotypes (70%) and environments were used to compose a drastically sparse training set (training environments/total of environments). This helped assess the efficiency of E-GP for Case 1, in which we were able to dissect the predictive ability in different scenarios of a scarcity of phenotypic records: novel genotypes in tested environments (*n*G, E), tested genotypes in untested environments (G, *n*E), and novel genotype and environment conditions (*n*G, *n*E). Tables 1, 2 present the results of N-level and multiregional sets, respectively. Then, these results were gathered for both the data sets and the four prediction scenarios in order to check for the analysis of joint predictive ability (Figure 3).

**Table 1:** Predictive ability (± standard error) of the genome-based prediction models (GP) for the N-level set of tropical maize hybrids (570 hybrids × 2 locations × 2 years × 2 nitrogen managements).

**Training setup**	**Model**	**Prediction Scenario**			
		**G, E**	**G, *n*E**	***n*** **G, E**	***n*** **G, *n*E**
7/8 environments	GBLUP	0.771 ± 0.064	0.397 ± 0.046	0.310 ± 0.054	0.297 ± 0.029
	E-GP (BD)	**0.903** ± 0.115	**0.493** ± 0.169	**0.615** ± 0.022	**0.416** ± 0.153
	E-GP (RN)	0.833 ± 0.118	**0.477** ± 0.199	**0.613** ± 0.040	0.394 ± 0.193
	W-GP (BD)	**0.915** ± 0.115	0.333 ± 0.208	**0.614** ± 0.025	0.242 ± 0.189
	W-GP (RN)	0.885 ± 0.117	0.327 ± 0.210	0.613 ± 0.031	0.230 ± 0.196
5/8 environments	GBLUP	0.747 ± 0.049	0.432 ± 0.046	0.294 ± 0.026	0.323 ± 0.04
	E-GP (BD)	0.905 ± 0.056	**0.554** ± 0.144	**0.659** ± 0.015	**0.464** ± 0.113
	E-GP (RN)	0.833 ± 0.056	**0.570** ± 0.132	**0.660** ± 0.025	**0.475** ± 0.104
	W-GP (BD)	**0.931** ± 0.057	0.449 ± 0.286	0.659 ± 0.019	0.347 ± 0.253
	W-GP (RN)	0.897 ± 0.056	0.501 ± 0.229	**0.660** ± 0.026	0.395 ± 0.198
3/8 environments	GBLUP	0.739 ± 0.040	0.527 ± 0.080	0.295 ± 0.015	0.394 ± 0.044
	E-GP (BD)	0.899 ± 0.026	0.534 ± 0.081	0.660 ± 0.012	0.388 ± 0.038
	E-GP (RN)	0.823 ± 0.026	**0.566** ± 0.086	**0.663** ± 0.015	**0.420** ± 0.041
	W-GP (BD)	**0.924** ± 0.026	0.532 ± 0.080	0.660 ± 0.015	0.384 ± 0.038
	W-GP (RN)	0.886 ± 0.025	**0.579** ± 0.088	**0.663** ± 0.020	**0.424** ± 0.041

*Values in bold denote higher predictive ability for each scenario: [G,E], known genotypes under known growing conditions; [G,nE], known genotypes under new growing conditions; [nG,E], new genotypes under known growing conditions; and [nG,nE], new genotypes under new growing conditions*.

**Table 2:** Predictive ability (± standard error) of the genome-based prediction models (GP) for the multi-local set of tropical maize hybrids (247 hybrids × 5 locations in different regions of Brazil).

**Training setup**	**Model**	**Prediction Scenario**			
		**G, E**	**G, *n*E**	***n*** **G, E**	***n*** **G, *n*E**
4/5 environments	GBLUP	0.953 ± 0.040	0.497 ± 0.072	0.552 ± 0.171	0.340 ± 0.138
	E-GP (BD)	**0.987** ± 0.006	**0.526** ± 0.054	**0.599** ± 0.097	**0.363** ± 0.131
	E-GP (RN)	0.873 ± 0.084	0.520 ± 0.064	0.496 ± 0.126	**0.358** ± 0.143
	W-GP (BD)	**0.989** ± 0.005	**0.527** ± 0.056	**0.599** ± 0.098	0.361 ± 0.131
	W-GP (RN)	0.931 ± 0.057	0.492 ± 0.078	0.501 ± 0.130	**0.366** ± 0.125
3/5 environments	GBLUP	0.927 ± 0.045	0.528 ± 0.066	0.543 ± 0.208	0.381 ± 0.142
	E-GP (BD)	**0.984** ± 0.006	**0.556** ± 0.052	**0.597** ± 0.097	**0.400** ± 0.131
	E-GP (RN)	0.845 ± 0.073	0.550 ± 0.059	0.477 ± 0.120	0.385 ± 0.135
	W-GP (BD)	**0.987** ± 0.005	**0.555** ± 0.053	**0.598** ± 0.095	**0.394** ± 0.132
	W-GP (RN)	0.915 ± 0.049	0.514 ± 0.072	0.483 ± 0.124	0.392 ± 0.119
2/5 environments	GBLUP	0.913 ± 0.050	0.552 ± 0.063	0.538 ± 0.223	0.409 ± 0.149
	E-GP (BD)	**0.982** ± 0.006	**0.574** ± 0.051	**0.593** ± 0.095	**0.410** ± 0.135
	E-GP (RN)	0.831 ± 0.069	0.572 ± 0.060	0.468 ± 0.117	0.394 ± 0.134
	W-GP (BD)	**0.986** ± 0.004	**0.575** ± 0.051	**0.592** ± 0.096	**0.411** ± 0.139
	W-GP (RN)	0.906 ± 0.046	0.539 ± 0.067	0.476 ± 0.119	0.404 ± 0.116

*Values in bold denote higher predictive ability for each scenario: [G,E], known genotypes under known growing conditions; [G,nE], known genotypes under new growing conditions; [nG,E], new genotypes under known growing conditions; and [nG,nE], new genotypes under new growing conditions*.

#### Within the Experimental Network

Predictions within known environmental conditions of a certain experimental network involve two scenarios: *G,E* and *nG,E*. For the *G,E* scenario (the classic “training set”), all the models outperformed the GBLUP in any setup of the N-level set and most of the setups of the multiregional set. The highest values of predictive ability were observed for enviromic-aided GP models using the block-diagonal matrix for G × E effects (BD), that is, the E-GP (BD) and W-GP (BD), respectively. Two general trends were observed: the size of the experimental setup has a small effect on the accuracy of the GP models. Second, higher accuracy gains were observed for the N-level set (Table 1), with a higher number of entries (more genotypes and more environments). The accuracy gains in the N-level set ranged from +8 (*r* = 0.83 for E-GP RN at 7/8 experimental setup), in relation to *r* = 0.77 (GBLUP), to +24% (*r* = 0.92 for W-GP RN at 3/8 experimental setup), in relation to *r* = 0.74 (GBLUP). In contrast, for the multiregional set (Table 2), both the RN-G × E models reduced the accuracy (on average by −3%). For the BD-G × E models, small gains in accuracy (from +4 to +8%) were observed.

That is also a trend for the second prediction scenario (*n*G, E), in which the multiregional set presented an average gain of 10% for all the enviromic-aided GP models with BD-G × E and a reduction of 10% for all the RN-G × E models. Conversely to the previous scenario (G, E, within the experimental network, using known genotypes), *n*G, E is one of the most important plants breeding scenarios. It represents the ability to predict new single-crosses within the known environmental gradient, by borrowing genomic and enviromic information from the phenotypes of the relatives, thus expanding the spectrum of possible genotypes using known growing conditions from the past. For the N-level set, gains of up to 100% were observed for all the enviromic-aided models using any G × E structure. No differences were observed between enviromic-aided models and experimental setups. On average, all the enviromic-aided models achieved a predictive ability of approximately *r* = 0.66 across all experimental setups (3/8, 5/8, and 7/8, Table 1). In contrast, the GBLUP model was impacted with reduced accuracy and a lack of phenotypic records. The highest gains in predictive ability were observed for scenario 3/8, with an average of +118% for the BD-G × E models, and +119% for the RN-G × E models.

#### Across the Experimental Network

The predictions of yet-to-be-seen growing conditions were evaluated by the scenarios *G, nE*, and *nG, nE*. For *G, nE*, the E-GP models outperformed W-GP and GBLUP across most of the experimental setups, despite small differences between the enviromic-aided approaches. For the E-GP (BD) at the N-level set (Table 1), the gains in predictive ability ranged from +24% (*r* = 0.49 in the 7/8 setup, Table 1), in relation to *r* = 0.4 (GBLUP), to +35% (*r* = 0.57 in the 5/8 setup), in relation to *r* = 0.43 (GBLUP). However, for scenario 3/8, the gains were equal to +10% (*r* = 0.57) in relation to the +13% archived by the benchmark W-GP (RN) (*r* = 0.58), both over the *r* = 0.53 from GBLUP. In scenario 7/8, W-GP was outperformed by GBLUP, with a reduction in accuracy between −18 and −16%, where the E-GP made better use of the large phenotypic information available for the training of the GP models (gains from +20 to +24% over GBLUP). A similar pattern was observed for the multiregional set (Table 2), in which the gains of E-GP ranged from +4 to +6% across all the setups, and W-GP ranged from −3 to +6% under the same conditions.

The *nG, nE* scenario is the most complex situation, because it is expected to predict yet-to-be-seen genotypes under unknown growing conditions. Thus, all the predictions were based on the quality of the association between the observed phenotypic records of relativities and their resemblance due to genomic or enviromic assembly. With a large size setup, it seems that the E-GP models outperform W-GP and GBLUP when predicting new G × E. Observed accuracy gains ranged from 33 (*r* = 0.39 for E-GP RN) to 40% (*r* = 0.42 for E-GP BD) in experimental setup 7/8 (Table 1), where GBLUP achieved *r* = 0.3, and from 47 (*r* = 0.46 for E-GP BD) to 51% (*r* = 0.48 for E-GP BD) in experimental setup 5/8, where GBLUP achieved *r* = 0.32. Unlike observations in the other prediction scenarios, the RN-G × E models outperformed BD-G × E in the following experimental setups: 3/8 (N-level set) and 2/5 (multiregional set).

### Accuracy Trends Across Diverse Experimental Setups

This section highlights the main target of our Case 1, in which the predictive ability was achieved using the merged information of scarce genotypes tested in some environments. Joint accuracy trends showed E-GP was useful for increasing GP accuracy (Figure 3A) and explaining the phenotypic variation sources in both maize data sets (Supplementary Tables 3, 4). For scenarios with reduced phenotypic information (e.g., 3/5, 3/8, and 4/8), any model with some degree of environmental information outperformed GBLUP in all the scenarios. The E-GP approach (purple colors in Figure 3A) better captured envirotype-phenotype relationships and converted them into accuracy gains among these models. This is also reflected in the efficiency of E-GP as a predictive breeding tool capable of reproducing field-based trials (Figure 3B). Regarding the G × E structures, the contribution of RN-G × E is significant only for drastically lacking phenotypic records (training setup 3/8), leading to the conclusion that the use of a main-effect is substantial and that, in most cases, E-GP is enough to increase accuracy in GBLUP. For setup 2/5 (multiregional set), no differences were observed among all the GP models.

The coincidence between the GP-based selection and the in-field selection (CS, %) ranged from ~35 to ~50%, in models with some environmental information, while it ranged between 30 and 40% for GBLUP (without environmental information). For the E-GP approach accounting for a wide number of phenotypic records in the training sets (7/8, 3/5, and 4/5), CS values of up to 55% were found. Among these models, it seems that RN-G × E reduces the CS estimates concerning the BD-G × E based models. Considering both Figures 3A,B, it is possible to suggest that predictive ability does not imply an increase in CS, that is, in the power of selecting the best performing genotypes in certain environments. However, the drastic increase in E-GP accuracy in relation to the other models leads us to infer that despite the lower rise in CS, the E-GP models are useful when predicting G × E for a vast number of single-crosses.

### Case 2: Enviromic Assembly With Optimized Training Sets for Genomic Prediction

The results mentioned above led us to investigate Case 2 (Figure 2), where we checked the possibility of training efficient and biologically accurate GP scenarios from super-optimized training sets. Then, we studied the potential of using these optimized field trials for predicting novel G × E under the so-called “virtual experimental networks.” This approach was implemented by combining two selective phenotyping approaches (Misztal, 2016; Akdemir and Isidro-Sánchez, 2019), aiming to identify combinations of genotypes and environments by *in-silico* representations of enviromic assembly × genomic kinships.

#### Predicting G × E in Virtual Experimental Networks

The process of designing virtual networks in maize hybrid breeding involved two steps (Supplementary Figure 1). First, we used a single-value decomposition (SVD)-based algorithm to select the effective number of individuals (*N*_GE_) (Misztal, 2016) representing at least 98% of the variation of **K**_*G,ET*_. It was done in **K**_*G,ET*_, because this kernel represents an *in-silico* representation of envirotypes and genotypes, which differs from the original approach that uses only genomic kinships (Akdemir and Isidro-Sánchez, 2019). Under sparse MET conditions, it led to a training size of *N*_GE_ = 67 and *N*_GE_ = 49 for the N-level (*n* = 4,560) and multiregional sets (*n* = 1,235), respectively. It represents only 1.5 and 4% of the whole experimental network (Supplementary Figures 2, 3). For didactic purposes, we will represent the values of *N*_GE_ as the training set size/number of genotypes from here onwards.

**Figure 4:**
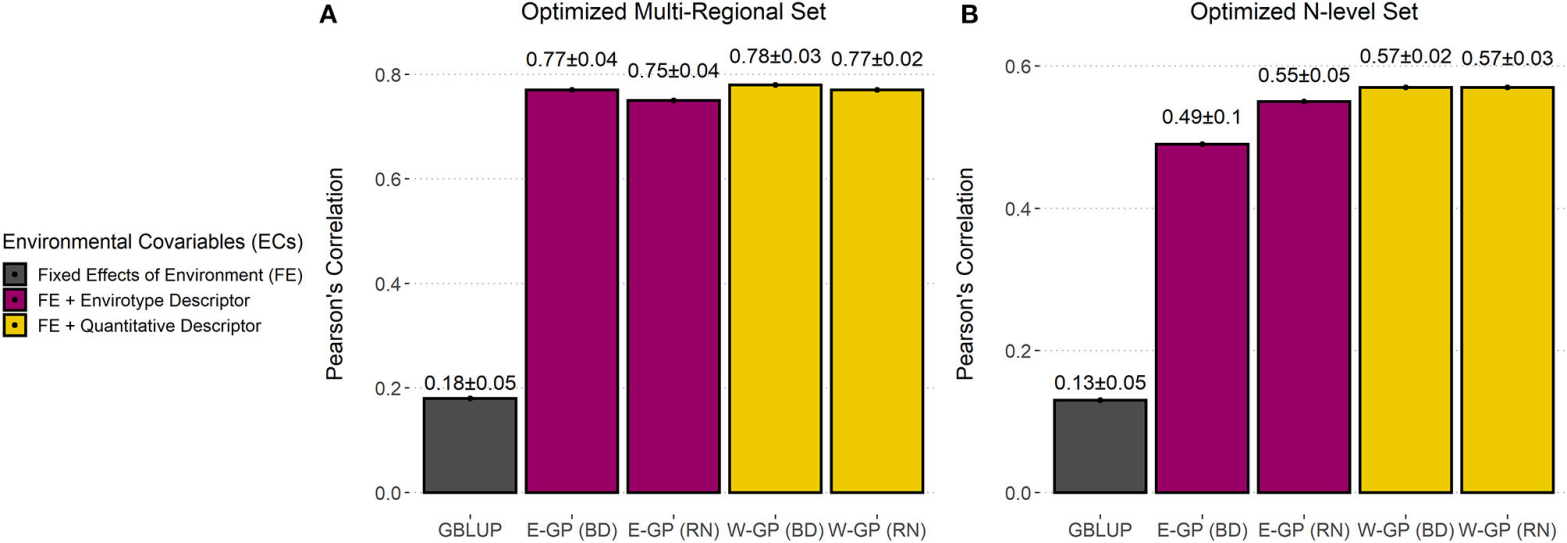
Accuracy of GP models trained with super-optimized experimental networks. Predictive ability (*r*) plus standard deviation measured by the correlation between observed and predicted values for each model in **(A)** the optimized multiregional set and for **(B)** the N-level set. Bar plots were colored according to the type of environmental covariable (ECs) used: none (black), envirotype descriptor (T matrix, wine), and quantitative descriptor (W matrix, yellow).

We also checked the use of all environments, although the differences in accuracy were small in relation to sparse MET scenario (Table 3). Furthermore, small differences were achieved by E-GP and W-GP models with BD-G × E, but both were higher than RN-G × E and GBLUP (Figure 4). Major differences were highlighted as follows. For within-field trials (observed phenotypes), the predictive ability ranged from *r* = 0.76 (W-GP) to *r* = 0.87 (E-GP). It was observed that lower values were due to lack of phenotypic records in the virtual networks, in which the predictive abilities ranged from *r* = 0.14 ± 0.11 (GBLUP) to *r* = 0.6 ± 0.06 (E-GP). However, in the virtual networks, it was observed that the predictive ability of models trained with drastically reduced phenotypic records ranged from *r* = 0.1 (GBLUP, N_GE_ = 67/4,560) to *r* = 0.58 (E-GP, N_GE_ = 67/4,560) and *r* = 0.18 (GBLUP, N_GE_ = 49/1,235) to *r* = 0.81 (E-GP, N_GE_ = 49/1,235). Therefore, it seems that the reduction of phenotype information does not compromise the enviromic-enriched models, but it is capable of delivering accurate predictions in some experimental networks, and in most cases, at least it will deliver consistent results with fewer phenotyping efforts.

**Table 3:** Predictive ability of the GP models for two tropical maize data sets (multiregional and N-level) produced using the effective number of phenotypic records (N_GE_, genotype-environment observations) and for the scenarios Field Trials (training set, that is; predicting N_GE_) and Virtual Network (predicting *n*–N_GE_, where *n* is the number of genotypes by environments available in the full data set).

**Scenario**	**Models**				
	**GBLUP**	**W-GP (BD)**	**W-GP (RN)**	**E-GP (BD)**	**E-GP (RN)**
**Multi-Regional set**					
**Field trials**					
N_GE_ = 210 (full)	0.698	0.962	0.892	0.964	0.893
N_GE_ = 210 (5%)	0.991	0.995	0.992	0.997	0.998
N_GE_ = 49 (full)	0.738	0.941	0.840	0.942	0.840
N_GE_ = 49 (5%)	0.991	0.991	0.991	1.000	1.000
**Virtual network**					
N_GE_ = 210 (full)	0.175	0.794	0.787	0.793	0.787
N_GE_ = 210 (5%)	0.098	0.736	0.750	0.713	0.715
N_GE_ = 49 (full)	0.190	0.810	0.788	0.810	0.789
N_GE_ = 49 (5%)	0.241	0.759	0.755	0.758	0.706
**N-Level set**					
**Field trials**					
N_GE_ = 536 (full)	0.982	0.984	0.775	0.991	0.775
N_GE_ = 536 (5%)	0.964	0.861	0.861	0.998	0.999
N_GE_ = 67 (full)	0.983	0.981	0.718	0.989	0.719
N_GE_ = 67 (5%)	0.967	0.833	0.802	0.998	1.000
**Virtual network**					
N_GE_ = 536 (full)	0.196	0.608	0.612	0.601	0.612
N_GE_ = 536 (5%)	0.152	0.554	0.545	0.406	0.484
N_GE_ = 67 (full)	0.102	0.574	0.572	0.578	0.573
N_GE_ = 67 (5%)	0.070	0.545	0.539	0.379	0.510

*The reference “full” and “5%” in parentheses represents the predictive ability produced with all genotypes using only the top 5%, respectively*.

The predictive ability was computed considering only the top 5% of genotypes in each environment and data set. The objective was to verify if the GP approaches could adequately predict the performance of the best-evaluated genotypes in the field. For the multiregional set, the predictive ability ranged from *r* = 0.098 (GBLUP, N_GE_ = 210/1,235) to *r* = 0.579 (W-GP BD, N_GE_ = 49/1,235) and *r* = 0.578 (E-GP BD, N_GE_ = 49/1,235); for the N-level set, W-GP outperformed E-GP, leading to *r* = 0.554 (W-GP BD, N_GE_ = 536/4,560) in front of *r* = 0.554 (E-GP RN, N_GE_ = 67/4,560) but with less phenotyping data. In contrast, the best E-GP model at the higher number of genotypes and environments evaluated in the field *r* = 0.484 (E-GP RN, N_GE_ = 536/4,560) was outperformed by the same model, but with less phenotyping data, *r* = 0.554 (E-GP RN, N_GE_ = 67/4,560). For GBLUP, the effective size of the training set was important, ranging in predictive ability from *r* = 0.07 (N_GE_ = 67/4,560) to *r* = 0.152 (N_GE_ = 536/4,560). The results of both sets suggest that when using enviromics-aided approaches, the use of a less but more representative amount of phenotyping information is better than the use of a higher but less representative number of phenotyping records collected across METs.

Figure 4 is created with the average values provided in Table 3 and shows that the optimization was more effective for growing conditions contrasting across macro-regions (Figure 4A) than for experimental networks involving fewer locations (Figure 4B). Notably, it is possible to drastically reduce field costs for experimental networks conducted across diverse locations.

#### Predicting Genotype-Specific Plasticity and Environmental Quality

**Figure 5:**
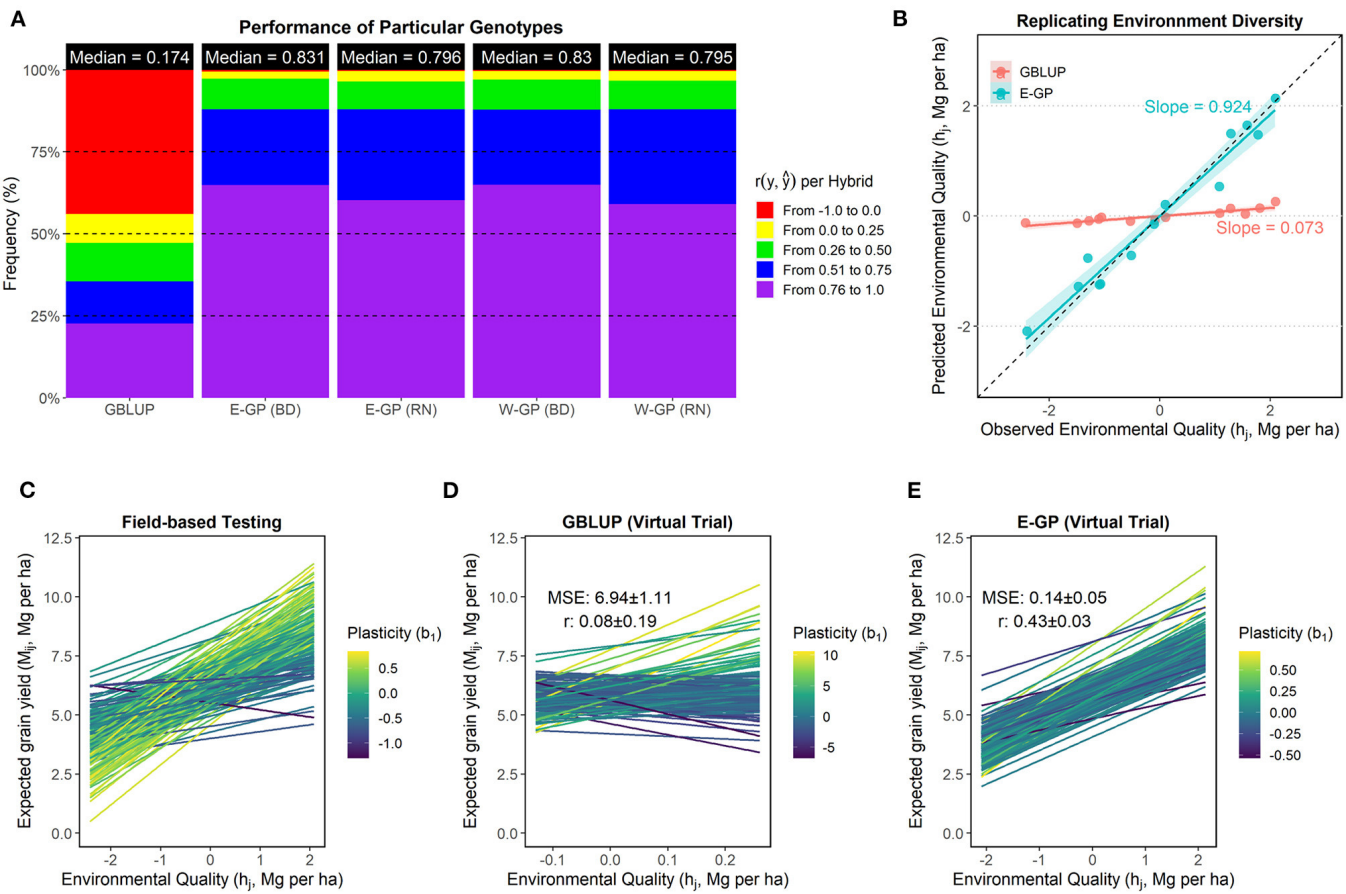
Accuracy of GP models in reproducing the genotype-specific plasticity. **(A)** Panel of predictive ability (*r*) explaining the plasticity of genotypes across environments. This statistic was estimated for each individual (hybrid) by correlating observed and predicted values across the environments. Individuals with values below 0 were considered unpredictable and marked in red. **(B)** Ability of the prediction-based tools to reproduce the quality of an existing experimental network (*h*_*j*_). In the X-axis, we computed *h*_*j*_ using the phenotypic records of a current experimental network. In the Y-axis, the *h*_j_ values are presented considering a virtual experimental network built using GBLUP and E-GP (with BD) predictions. **(C–E)** Yield plasticity panels denoting G × E effects of each genotype across the *h*_*j*_ values for observed field testing screening **(C)** concerning prediction-based **(D,E)**. Only the 5% best genotypes in each environment were used to create this plot. Each line was colored with the genotype-specific plasticity coefficient (*b*_1_). For the N-level set, the full-optimized set (536 hybrids over eight environments) was used.

In this step, we checked the ability of the models to perform virtual screenings for yield plasticity (Figure 5). We used the Finlay–Wilkinson method (FW, Equation 7) over the predicted GY means of each genotype *i* in environment *j* (*M*_*ij*_). Hence, we compared the ability of E-GP in the prediction of (1) individual genotypic responses across environments, (2) the gradient of environmental quality (*h*_*j*_), and (3) the plasticity coefficient (*b*_1_) of the FW model describing the rate of responsiveness to *h*. The results shown in Figure 5 involve a joint analysis of both data sets.

All models that included some degree of enviromic assembly outperformed the GBLUP-based approach when predicting individual genotype responses across the METs (Figure 5A). The median values of *r* ranged from 0.17 (GBLUP), in which 45% of the genotypes were not well-predicted (red colors), to 0.83 (E-GP), in which up to 60% of the genotypes were very well-predicted (purple colors). The inclusion of any enviromic assembly and G × E structure led to drastic gains in accuracy for a particular genotype response across contrasting (and unknown) G × E conditions (gains up to ~378%). However, the BD structure outperformed RN in resolution (many purple colors in Figure 5A). A major part of the accurately predicted performance of genotypes across the environments ranged from *r* = 0.75 to *r* = 1. Due to this, for the next figures, we plotted only the E-GP considering the BD-G × E structure.

The GBLUP approach was unable to correctly reproduce *h*_*j*_ for an *in silico* study using the FW model (Figure 5B). We observe that E-GP better describes the *h*_*j*_ gradient (mean-centered average values of GY for each environment), with *r* close to 1 (correlation between observed and predicted environmental qualities), also suggesting a low bias (slope = 0.924 between observed and predicted values). Consequently, this was reflected in the quality of yield plasticity predictions (Figures 5C–E), as yield plasticity was represented as linear responsiveness over the environmental variation. The graphical representation of genotype-specific linear reaction norms dictated by the linear regression slope (*b*_1_) was likely more similar to E-GP than GBLUP to those observed in field-based testing (Figure 5B). The accuracy of *b*_1_ ranged from *r* =0.08 (GBLUP) to *r* = 0.43 (E-GP), with an increase of 437%.

## Discussion

Large-scale envirotyping, or simply enviromics, is an emerging field of data science in agricultural research and modern breeding program routines. Here, we demonstrated that enviromics is capable of bringing together environment information and quantitative genomics in an ecophysiology-smart manner. In this study, we presented the first report on (1) the use of Shelford's Law to guide the assembly of the enviromics for predictive breeding purposes over experimental networks; (2) the integration of enviromic assembly-based kernels with genomic kinship into optimization algorithms capable of designing selective phenotyping strategies; (3) a break of the paradigm relying on the fact that phenotyping a higher number of genotypes in a higher number of environments does not always contribute to increasing the accuracy of GP for contrasting G × E scenarios, but there are pieces of evidence suggesting that enviromics increases accuracy in sparse multi-environment networks; and (4) the process of deriving markers of environmental relatedness, here called “enviromic assembly,” that is crucial for the implementation of low-cost GP platforms under multi-environmental conditions.

In this study, we also envisage that the process of enviromic assembly is supported by a strong theoretical background in ecophysiology, illustrating the potential uses of environmental information to increase the accuracy of predictive breeding for yield and plasticity. Our results indicate that the E-GP platform (Figure 2) can fit two types of prediction scenarios in plant breeding programs: (1) better use of available phenotypic records to train more accurate GP models capable of aiding the selection of genotypes across multi-environmental conditions, and (2) a method that reduces costs for field-based testing and enables early screening for yield plasticity under crossover G × E conditions. Furthermore, we show that any model with some degree of enviromic assembly (by typology or quantitative descriptors) is always better in reproducing the environmental quality of genotypes in field trials and phenotypic plasticity.

Below, we discuss the aspects that support the use of E-GP for multi-environment predictions, involving the importance of breaking the paradigm that states that enviromics are not necessary to predict G × E accurately. We then discuss how genomic and enviromics sources are linked in the phenotypic records collected from the fields and how this knowledge can improve the quality of prediction-based pipelines for crop improvement. Finally, we envisage possible environmental assembly applications supporting other predictive breeding fields, such as optimizing crop modeling calibration, and how it can couple with a novel level of climate-smart solutions for crop improvement by anticipating the plasticity of many genotypes using reduced phenotypic data.

### Benefits of Enviromics for Multi-Environment Genomic Prediction

Genomic prediction platforms were first designed to model genotype-to-phenotype relationships under single environment conditions, e.g., in a breeding program nursery (Lorenzana and Bernardo, 2009; Windhausen et al., 2012; Zhao et al., 2012; Zhang et al., 2015). Under these conditions, the micro-environmental variations within breeding trials (e.g., spatial gradients in soil properties) are minimized in the phenotypic correction step by separating useful genetic patterns and experimental noises (non-genetic patterns). However, the phenotypic records carry the indissoluble effects of macro-environmental fluctuations of certain weather and soil factors that occurred during crop growth and development (Li et al., 2018; Millet et al., 2019; Vidotti et al., 2019; Guo et al., 2020; Jarquin et al., 2020). This seems to be of no concern when predicting novel genotypes under the same growth conditions (the CV1 scheme for single-environment models) but becomes noise for multi-environment prediction scenarios. It is a consequence of macro-environment fluctuations in the lifetime of crops (Allard and Bradshaw, 1964; Bradshaw, 1965; Arnold et al., 2019), responsible for modulating the rate of gene expression (e.g., Jończyk et al., 2017; Liu et al., 2020), fine-tuning epigenetic variations and transcriptional responses (e.g., Vendramin et al., 2020; Cimen et al., 2021).

For each unit that we call “environment” (field trial in a specific year, location, planting date, and crop management), there are various environmental factors, such as water availability, canopy temperature, global solar radiation, and nutrient content in the soil. The balance of these conditions will dictate the availability of resources for each crop species across each development stage, that is, considering its physiological specificities, which is a consequence of the aforementioned environmental growing conditions and the current phenotypic architecture in the soil-plant-atmosphere momentum. Thus, for each crop species, with a different phenotypic architecture of roots, leaves, and accumulated biomass, it is also expected that the quality of certain environment will also have a range because of species-specific sensibilities. This is one of the main principles for conceiving crop growth models since the establishment of the “School of de Wit” back in the 1960s (see Bouman et al., 1996). However, it also seems very likely with a previous theory in ecophysiology, which suggests that the fitness of a population is given by the amount and distribution of resources available for its establishment and adaptation (Shelford, 1931). Thus, we reinterpret this concept by assuming that the relation between input availability (deficit, optimum amount, or excess), across different crop development stages, drives the rate and amount of the genetic potential for a given environment. Therefore, it provides the foundations to elaborate the argument that there is also an indissoluble envirotype-phenotype covariance in the phenotypic records that is interpreted as a G × E interaction for each environment. Because of that, we envisage that any environmental relatedness kernel must account for it in any way, for it seems that the typology-based matrix is the more biological accurate way to parametrize environmental information aimed for enviromic assembly.

The pioneer approaches to measuring crop adaptability use the average value of a given trait in each environment as an environmental quality index (e.g., Finlay and Wilkinson, 1963). However, the problem with this approach is that it explains the quality of the environment realized by the genotypes evaluated in it, making it inefficient in explaining the drivers of environmental quality and incapable of predicting untested growing conditions, as observed in our results for Case 2 using GBLUP without enviromic data. In addition, our results for Case 1 highlight that there is a limit in accuracy for traditional GBLUP across METs, in which the accuracy remains almost the same, regardless of the number of phenotypic records available.

A second intrinsic covariance can interpret the last result within the phenotypic records, which is the genotype-envirotype covariance. By adapting the Quantitative Genetics theory to the terminology used here, we can infer that each genotype reacts differently to each envirotype, resulting in each phenotype. This phenotype is then used to provide small crop phenology differences (genetically determined window sizes for each development stage). Recent but pioneer studies were carried out to understand the genetic and environmental determinants of flowering time in sorghum (Li et al., 2018) and rice (Guo et al., 2020) that can be indirectly interpreted as cardinal differential thresholds for temperature response. Furthermore, Jarquin et al. (2020) proved that it is possible to increase the ability of genomic prediction (GP) in predictive novel G × E by coupling information of day-length in benchmark GP models. For all the examples reported above, we can infer that, when trying to predict a novel genotype, borrowing genotypic information from the relatives in different environments makes it impossible to reproduce the genotype-envirotype covariance without adding any enviromic information into the model.

The presence of both genotype-envirotype and envirotype-phenotype covariances might explain the gains in the predictive ability due to the use of multi-environment GP models in contrast to single-environment GP models (Bandeira e Souza et al., 2017; de Oliveira et al., 2020) and why deep learning approaches have successfully captured intrinsic G × E patterns and translated them into gains in accuracy (Montesinos-López et al., 2018; Crossa et al., 2019; Cuevas et al., 2019). Conversely, this also might explain the need to incorporate secondary sources of information in the prediction of grain yields across multiple environments (Westhues et al., 2017; Ly et al., 2018; Millet et al., 2019; Jarquin et al., 2020; Costa-Neto et al., 2021a,b), as well as the possible limitations of CGM approaches contrasting scenarios differing from those targeted near-iso conditions of CGM calibration (e.g., Cooper et al., 2016; Messina et al., 2018). Thus, an alternative could be supervised approaches to describe the environmental relatedness, such as in this article, and perhaps unsupervised algorithms capable of taking advantage of the covariances related to genotype-phenotype, genotype-envirotype, and envirotype-phenotype dynamics.

### Sometimes Main-Effect Enviromics Is Better Than Reaction Norm Models

Our results from Case 1 show that the inclusion of enviromic sources (for main effects or explicitly incorporated in the RN-G × E structure) led to a better description of the envirotype-phenotype covariances, which was reflected in accuracy gains. Based on our data and the Bayesian approach used, it is worth highlighting that incorporating enviromic sources does not replace the incorporation of a design matrix for environments (here used as fixed effects), as is commonly associated in previous studies of GP reaction norms. Here, we show that enviromic sources came up as tentative to capture the envirotype-phenotype covariances. The cross-validation scheme used in Case 1 allowed us to observe that the joint prediction of different genotype-environment conditions (Figure 3) might highlight better how enviromic sources can contribute to increasing the predictive ability of GP, mostly due to its usefulness in approaching the environmental correlation among field trials. Furthermore, it shows more transparency for the influence of scenarios G, *n*E, and *n*G *n*E, in which we had a considerable lack of phenotypic information on training GP. Thus, we can infer that schemes such as CV1 (only *n*G, E) are the least adequate option to show the benefits of coupling enviromics in GBLUP. However, looking at a drastically sparse MET condition (joint prediction scenarios) shows that enviromics improves the accuracy of GP as the size of the MET also increases. Predictions are made up of tiny experimental networks.

### Differences Between W and T Covariable Matrices

Regarding the enviromic assembly approaches used in this study, there was evidence that using typologies as envirotype descriptors (T matrix) is more biologically accurate in representing environmental relatedness than using quantitative descriptors (W matrix) based on quantile covariables. The use of typologies directly integrates the classic approaches used for environmental characterization (e.g., Chenu et al., 2011; Heinemann et al., 2015, 2019), thus providing a single platform to integrate historical studies of environmental impacts in the design of environmental relationship matrices for predictive purposes. This represents an increase in the biological accuracy of the GP models, which can also reflect in the statistical accuracy. In conclusion, it can boost the ability of plant breeders to better select and recommend cultivars across multi-environment conditions. Further efforts in this sense must be devoted to increase the level of explanation of the genotype-envirotype covariances, which can also take advantage of Shelford's Law to refine the limits of tolerance for genotypes. Thus, different genotypes will be under the influence of a diverse set of envirotypes, which can be realized for the same environmental factor (e.g., solar radiation, air temperature, soil moisture) according to its occurrence across crop lifetime (e.g., vegetative stage) and the adaptation zone designed from ecophysiology concepts (e.g., temperature cardinals defining which temperature level results in stress and optimum growing conditions). Because of that, we envisage that further studies must be conducted to create a genotype-envirotype × environment T matrix, that is, a matrix considering genotype-specific envirotypes also based on genotype-specific cardinal thresholds and tolerance limits for discriminating each typology of adaptation.

A second difference may be explained because quantitative environmental covariates are not an additive effect to compose an environment variation. Despite this, we agree with Resende et al. (2021), and we adapted the idea of envirotypes as markers of environment-relatedness differently. For example, the common use of mean values of covariates, such as rainfall, solar radiation, and air temperature, represents a non-additive between each other; however, they are very well-correlated for a given site-planting date condition, even when using strategies to deal with collinearity, such as partial least squares (e.g., Vargas et al., 2006; Porker et al., 2020). We can use as an example a given day of crop growing when a large amount of rainfall has occurred. We can suppose that the sky is cloudy, with less radiation and lower temperature. Thus, using such a G-BLUP inspired approach is not an ideal solution to estimate the environmental variance. Conversely, environmental typologies (T) are based on frequencies (ranging from 0 to 1), where the sum of all frequencies is equal to 1 (100% of the variation). In addition, these typologies can be built for a given site using historical weather data, adapting the approach of Gillberg et al. (2019) and de los Campos et al. (2020). As presented in section GBLUP with enviromic main effects from T matrix (E-GP), if no typologies are considered, the expected environment effect is given for a fixed-environment intercept (with 0 variances within and between environments). Despite this fact, another option is using non-linear kernel methods to estimate only the environment-relatedness, as this approach takes advantage of non-linear relationships among covariates (Costa-Neto et al., 2021a,b).

### Does More Phenotype Data Mean Higher Accuracy in Multi-Environment Prediction?

This study shows that environmental information can break the paradigm that claims that more phenotype information leads to higher accuracy of GP models over METs. Our results highlight that the traditional GBLUP models assume that the variation due to G × E is purely genomic-based across field trials, leading to an implicit conclusion that the yield plasticity is constant (slope ~ 0) for all genotypes, which is unrealistic. It also reflects that G × E patterns are non-crossover (scale changes in performance across different variations), that is, a well-performing genotype will always be good across environments, and a poorly performing genotype has the same trend for all environments. Despite the gains achieved in predicting the quality of a novel environment and the plasticity for tested and untested genotypes, we noticed that the inclusion of enviromic sources also leads to the unrealistic conclusion that all genotypes respond in the same way gradient of climate and soil quality. Our results show a reasonable accuracy in predicting yield plasticity, but further efforts must be made to improve the explanation by this approach of yield plasticity as a non-linear variation across the gradient of environmental factors. In addition, further studies using larger experimental networks and other crop species must be conducted to check the consistency of the suggestions we made from our proof-of-concept study. Finally, if the experimental network is based on a higher number of environments, perhaps the use of enviromic assembly will also serve to find groups of mega-environments that historically share the same typology. Then, the G × E optimization algorithm would be used within each historical mega-environment.

We observed that the use of selective phenotyping strategies made with enviromic assembly × genomic kinships showed a drastic reduction of in-field efforts. Furthermore, combined with enviromic-aided GBLUP models, it led to the development of almost the same predictive ability, using a large number of genotypes and environments for a large experimental network. Thus, we can enumerate the benefits of the enviromic-based approaches tested in this study as: (1) the possibility of training prediction models for yield plasticity with reduced phenotyping efforts, (2) the assembly of enviromics with genomics allowing the selection of the genotype-environment combinations and the envirotype-phenotype covariances among phenotypes across different environments.

Considering both enviromics approaches evaluated in this study, we conclude that the advantages of E-GP over W-GP can be enumerated as (1) the flexibility to design a wide number of the environment types assuming different frequencies of occurrence of key stressful factors in crop development; (2) it allows the use of historical weather and in-field records to compute trends of certain envirotypes in certain environments, which can be coupled into (3) the definition of TPE and characterization of mega-environments, as the main approach used for this relies on studies on the frequency of occurrence of the main environment types (e.g., Heinemann et al., 2019). For the latter, for example, the T matrix proposed here is just an arrangement of an environment × typology matrix, in which each entry represents its frequency of occurrence at a particular time interval of the crop lifetime. Conversely, the advantages of W-GP over E-GP rely on plasticity in creating large-scale envirotype descriptors with reasonable biological accuracy. Finally, it does not depend on other steps for checking covariate importance and any criteria of covariate selection, because the all-possible environmental information, with some ecophysiological sense, will be used and discretized in typologies by literature-based criteria (e.g., cardinals of temperature), agronomic expertise, or statistical criteria (distribution tables). Then, the resemblance between two different field trials will be approached based on the shared frequencies of the possible typologies for your crop and germplasm. Finally, selective phenotyping will be guided by shared typologies, not by quantitative relationships within the collected environmental information.

### Can We Envisage Climate-Smart Solutions From Enviromics With Genomics?

Modern plant breeding programs must deliver climate-smart solutions cost-effectively and time-reduced (Crossa et al., 2021). By climate-smart solutions, we mean (1) adopting cost-effective approaches capable of providing fast and cheap solutions to face climate change, (2) better resource allocation for field trial efforts to collect representative phenotype information to feed prediction-based platforms for crop improvement, such as training accurate GP models and CGM-based approaches capable of guiding several breeding decisions, (3) a better understanding of which envirotypes most limit the adaptation of crops across the breeding TPE, revising historical trends and expecting future scenarios (e.g., Ramirez-Villegas et al., 2018, 2020; Heinemann et al., 2019), and (4) understanding the relationship between secondary traits and their importance in explaining the plant-environment dynamics for given germplasm at a given TPE (e.g., Cooper et al., 2021). However, most of these objectives will be hampered if MET-GP platforms do not consider models with a higher biological meaning (Hammer et al., 2019) and reliable environmental information. A cost-effective solution for that, if the breeder has no access to sensor network tools, relies on the use of remote sensing tools to collect and process historical weather data (from 1981 to present) and other sources of data (soil data, such as SoilGrid, https://soilgrids.org/) using tools from the open-source EnvRtype R package (Costa-Neto et al., 2021b). However, this data source is only efficient for field trials with a certain geographic distance, as the current NASA POWER resolution is 1/2 by 1/2 arc degree (almost 0.5 ×0.5 degrees of latitude × longitude), which was the case of our data set in this study.

Suppose selective phenotyping is added in the enviromics-aided pipeline for GP (Supplementary Figure 1). In that case, additional traits and the possibility of screening genotypes across a wide number of managed environments will increase. Furthermore, it can support the training of field trials for CGM approaches, which demand the phenotyping of traits across crop lifetime, such as biomass accumulation and partitioning among different plant organs. Finally, using models considering an explicit environmental gradient of key environmental factors is a second alternative for this approach. It can be done to discover the genetic determinants of the interplay between plant plasticity and environmental variation. As a wide range of genes reacts to each gradient of environmental factors, the use of whole-genome regressions of reaction norms for each environmental factor must be useful to screen potential genotypes (in our case, single-crosses) for a diverse set of scenarios (e.g., increased heat stress). Pioneer studies used this methodology in wheat breeding (Heslot et al., 2014; Ly et al., 2018) and inspired other cereal crop applications.

For example, Millet et al. (2019) developed an approach including whole-genome regressions and factorial regression for the main environmental drivers of G × E. In the past, studies involving FR analysis found that the effect of high temperatures on grain filling and maturation (Epinat-Le Signor et al., 2001; Romay et al., 2010), water balance at flowering (Epinat-Le Signor et al., 2001; Millet et al., 2019) and intercept radiation in the vegetative phase (Millet et al., 2019) are the main drivers of G × E for yield components in maize. Thus, Millet et al. (2019) explored this opportunity offered by FR to use genotypic-specific regressions, which coupled with genomic data, led to an increase of 55% in the accuracy of MET-GP over the benchmark environmental similarity model made of mean values of environmental factors, as proposed by Jarquín et al. (2014).

From the aspects mentioned above, we envisage that the use of GP for multi-environment predictions must account for some degree of ecophysiological reality while also considering the balance and relationship between parsimony and accuracy (Hammer et al., 2019; Cooper et al., 2021; Costa-Neto et al., 2021b). Here, we also highlight that multi-environment GP must account for the impact of (1) resource availability in creating biologically accurate platforms in training CGM-based approaches and delivering reliable envirotyping information for those purposes, and (2) the availability of the knowledge of experts in training CGM approaches. Thus, ecophysiology concepts to provide solutions for raw environmental data processing in enviromic assembly information for predictive purposes seem to be a cost-effective alternative to leverage accuracy involving parsimony and biological reality.

## Data Availability Statement

The original contributions presented in the study are included in the article/Supplementary Material, further inquiries can be directed to the corresponding author/s.

## Author Contributions

GC-N conceived the theory, performed the data analysis, and wrote the manuscript. RF-N generated the dataset. RF-N and JC revised the text, figures, and tables, which all the authors finally edited.

## Funding

Open Access fees are received from the Bill and Melinda Gates Foundation. We acknowledge the financial support provided by the Bill and Melinda Gates Foundation [INV-003439 BMGF/FCDO Accelerating Genetic Gains in Maize and Wheat for Improved Livelihoods (AG2MW)] as well as USAID projects [Amend. No. 9 MTO 069033, USAID-CIMMYT Wheat/AGGMW, AGG-Maize Supplementary Project, AGG (Stress Tolerant Maize for Africa)] that generated the CIMMYT data analyzed in this study. We are also thankful for the financial support provided by the Foundations for Research Levy on Agricultural Products (F.F.J.) and the Agricultural Agreement Research Fund (J.A.) in Norway through NFR grant 267806 as well as the CIMMYT CRP (maize and wheat).

## Conflict of Interest

The authors declare that the research was conducted in the absence of any commercial or financial relationships that could be construed as a potential conflict of interest. The reviewer KD declared a shared affiliation, with no collaboration, with one of the authors GC-N to the handling editor at the time of the review.

## Publisher's Note

All claims expressed in this article are solely those of the authors and do not necessarily represent those of their affiliated organizations, or those of the publisher, the editors and the reviewers. Any product that may be evaluated in this article, or claim that may be made by its manufacturer, is not guaranteed or endorsed by the publisher.

## Abbreviations

A: Additive effects
b: Coefficient of yield adaptability from Finlay–Wilkinson
BD: Block-diagonal matrix of the genomic by environment effect
D: Dominance effects
EC: Environmental covariate
E-GP: Enviromic-aided genomic prediction including envirotype markers
Envirotype: Environmental-type
FW: Finlay–Wilkinson adaptability model
GBLUP: Genomic best linear unbiased predictions
G × E: Genotype by environment interaction
GP: Genomic prediction
MET: Multi-environment trials
MSE: Mean squared error
N_GE_: Minimum core of genotype-environment combinations
OTS: Optimized training sets for genomic prediction
r: Predictive ability given by the average linear correlation between observed and predicted trait values
RN: Enviromic by genomic matrix for reaction norm effects
T: Typology matrix of envirotype markers (qualitative covariables and their frequencies)
W: Environmental covariable matrix (quantitative covariables)
W-GP: Enviromic-aided genomic prediction using quantitative environmental covariates.
