# Supplementary material for: Enviromic Assembly Increases Accuracy and Reduces Costs of the Genomic Prediction for Yield Plasticity in Maize
Source: Front Plant Sci. 2021 Oct 7;12:717552. doi: 10.3389/fpls.2021.717552 (PMC8529011; doi:10.3389/fpls.2021.717552)
Supplement: Supplementary file 1 [file Data_Sheet_1.PDF]

Supplementary Material

1 Supplementary Tables

Supplementary Table 1. Geographic coordinates of each experimental site and environments used in Multi-Regional Set in 2015, South America, Brazil.

ID	Site	Region	Year	Management	Latitude	Longitude
NM	Nova Mutum	Middle-Western	2015	all necessary management practices to achieve Potential Yield	-13.05	-56.05
SO	Sorriso				-12.32	-55.42
PM	Patos de Minas	Southwestern			-18.34	-46.31
IP	Ipiaçú				-18.9	-49.56
SE	Sertanópolis	South			-23.03	-51.02

Supplementary Table 2. Geographic coordinates of each experimental site and environments used in N-level set between 2016 and 2017 in South America, Brazil.

ID	Site	Region	Year	Management	Latitude	Longitude
1_PI_LN	Piracicaba	Southwestern	2016	Low N Level	-22.705	-47.637
1_PI_IN				Ideal N Level	-22.705	-47.637
1_AN_LN	Anhumas			Low N Level	-22.87	-47.997
1_AN_IN				Ideal N Level	-22.87	-47.997
2_PI_LN	Piracicaba		2017	Low N Level	-22.705	-47.637
2_PI_IN				Ideal N Level	-22.705	-47.637
2_AN_LN	Anhumas			Low N Level	-22.87	-47.997
2_AN_IN				Ideal N Level	-22.87	-47.997

Supplementary Table 3. Estimated variance components (±standard deviation) for N-level set.

Effect	Model				
	GBLUP	W-GP (BD)	E-GP (BD)	W-GP (RN)	E-GP (RN)
T	-	-	0.195±0.016	-	0.324±0.029
W	-	0.319±0.029	-	0.643±0.06	-
A	1.371±0.031	1.392±0.03	1.350±0.03	1.330±0.029	1.396±0.036
D	0.574±0.005	0.575±0.005	0.575±0.005	0.550±0.005	0.426±0.005
AE	0.363±0.003	0.366±0.004	0.365±0.003	-	-
DE	0.337±0.003	0.338±0.003	0.336±0.003	-	-
AT	-	-	-	-	0.034±0
AW	-	-	-	0.016±0.001	-
DT	-	-	-	-	0.012±0
DW	-	-	-	0.006±0.002	-
Residual	1.154±0.004	1.153±0.004	1.155±0.004	1.45±0.003	1.37±0.003
Explained variance (%)	70%	72%	71%	64%	62%

Supplementary Table 4. Estimated variance components (±standard deviation) for Multi-Regional Set.

Effect	Model				
	GBLUP	W-GP (BD)	E-GP (BD)	W-GP (RN)	E-GP (RN)
T	-	-	0.157±0.019	-	0.055±0.004
W	-	0.090±0.007	-	0.078±0.007	-
A	0.464±0.012	0.465±0.012	0.464±0.011	0.462±0.011	0.499±0.014
D	0.218±0.004	0.217±0.004	0.216±0.004	0.211±0.004	0.232±0.005
AE	0.197±0.003	0.199±0.003	0.195±0.003	-	-
DE	0.089±0.001	0.088±0.001	0.089±0.001	-	-
AT	-	-	-	-	0.007±0.009
AW	-	-	-	0.004±0.002	-
DT	-	-	-	-	0.002±0.001
DW	-	-	-	0.002±0.003	-
Residual	0.234±0.001	0.235±0.001	0.235±0.001	0.239±0.001	0.237±0.001
Explained variance (%)	81%	82%	83%	76%	77%

2 Supplementary Figures

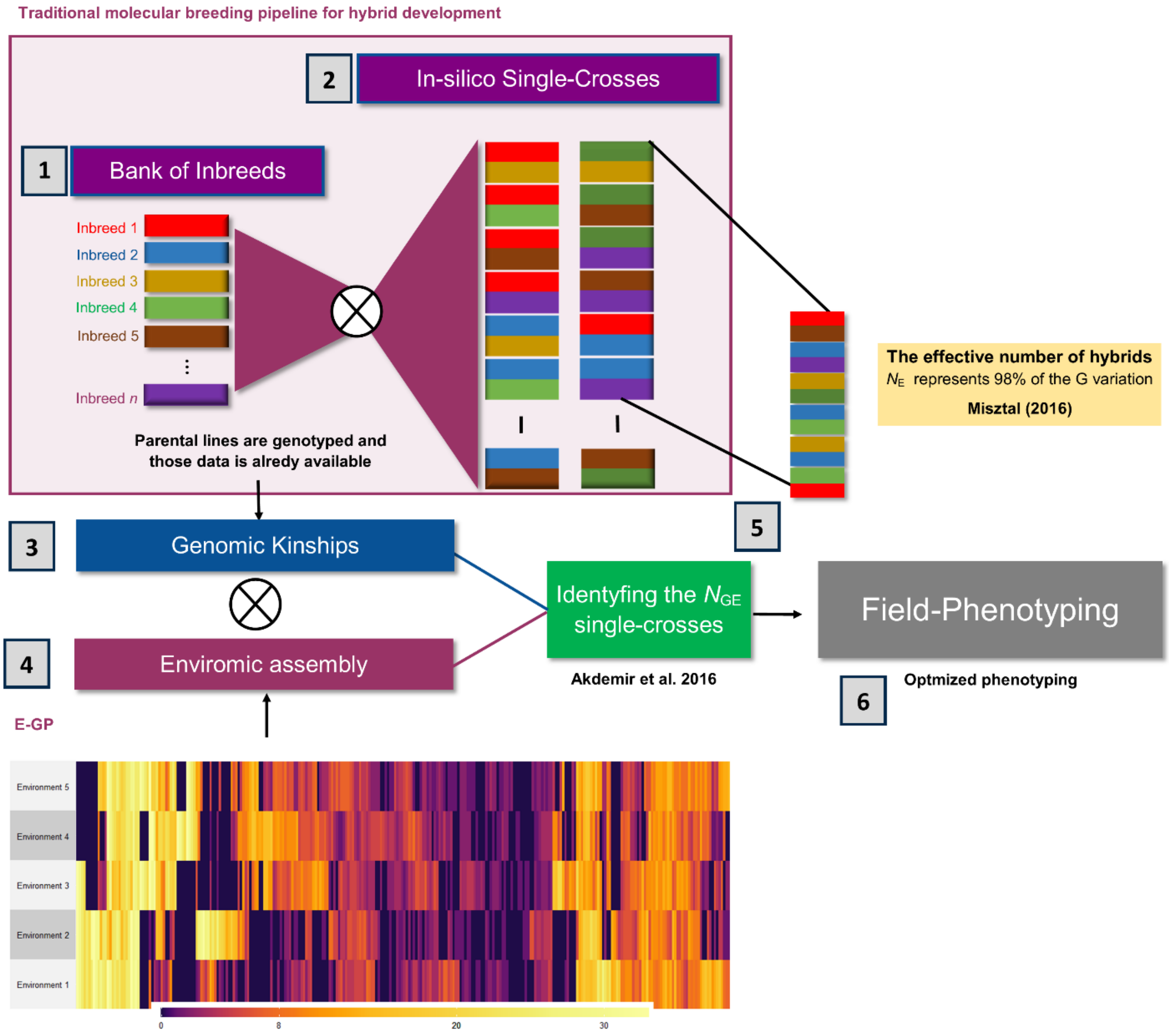

Supplementary Figure 1. Use of selective phenotyping with genomic kinship and enviromic assembly for boosting the hybrid breeding pipelines. In purple, it is presented the current molecular breeding approaches of hybrid development. (1-2) **(1)** From a bank of elite inbreeds already genotyped, it is possible to create a large number of in-silico single-crosses **(2)** using the Kronecker product between each SNP marker from desirable parentals. Then, a single-value decomposition (SVD) of the genomic kinship (G) reveals the effective number of genotypes (N_E) that represents at least 98% of the variation in G **(3)**. However, under the E-GP platform, the use of G plus an enviromic assembly (T) for a target population of environments (TPE) can be used to build up in silico possible growing conditions that crops may experience **(4)**. Then, via the Kronecker product between enviromic x genomic, it is possible to build a matrix accounting for genotypic observations per environment. Then, we apply SVD on that to select the effective number of genotypes per environments (N_{GE}) that represents at least 98% of the variation of the realized experimental network **(5)**. Later, using the

SPTGA package, that provides a genetic algorithm, we can define the most relevant combinations of genotype x environment **(6)**. Finally, only these individuals are phenotyped in specific locations. Then, it will be used as a training population for genomic-based prediction or other research purposes, such as training crop growth models or running a factorial regression analysis. This approach allows an optimized training of those models, which may increase efficiency in predicting phenotypic landscapes across novel growing conditions

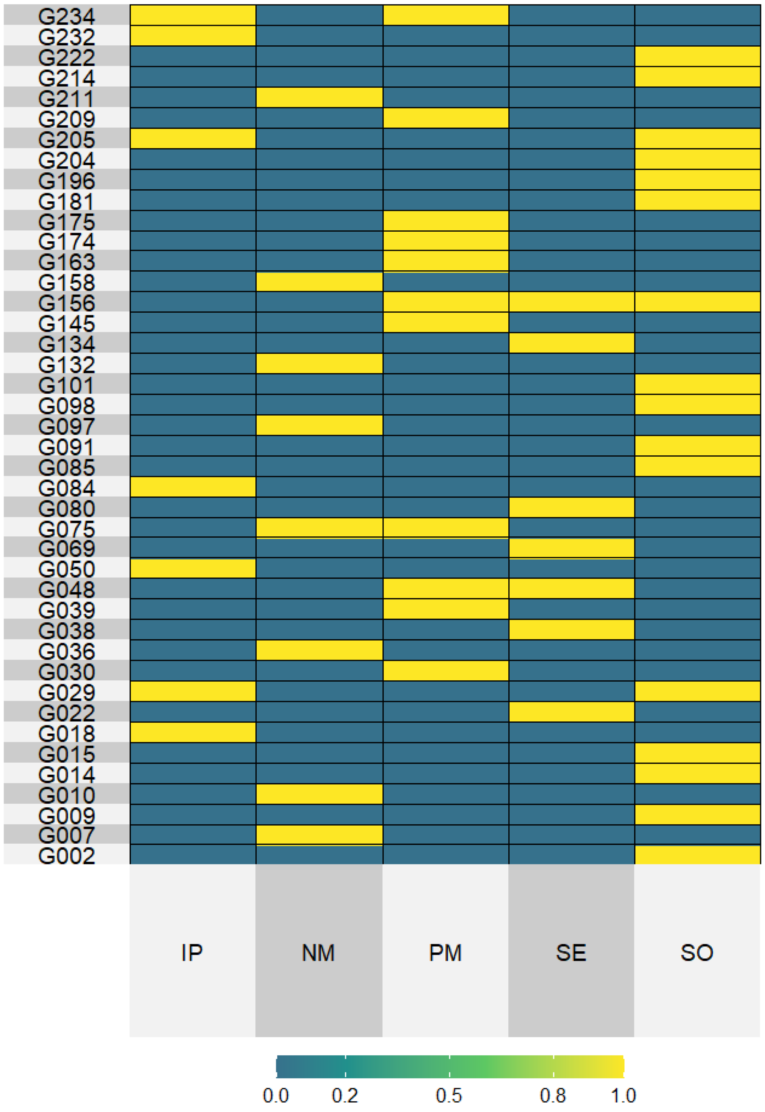

Supplementary Figure 2. Summary of the selective phenotyping approach drawn by the effective number of observations (N_{GE}) phenotyped in the field for the Multi-Regional Set (247 tropical maize hybrids over 5 locations). The core of 42 maize hybrids (rows) per 5 environments (columns). In yellow is the hybrid-environment combinations phenotyped in the field-based trials. It resulted in N_{GE} = 49, because some genotypes occur in more than one environment. At each environment, the number of genotypes was: IP (7), NM(8), PM (11), SE (7), and SO (16). The

remaining 205 hybrids plus the blue cells were considered a testing set (virtual experimental network).

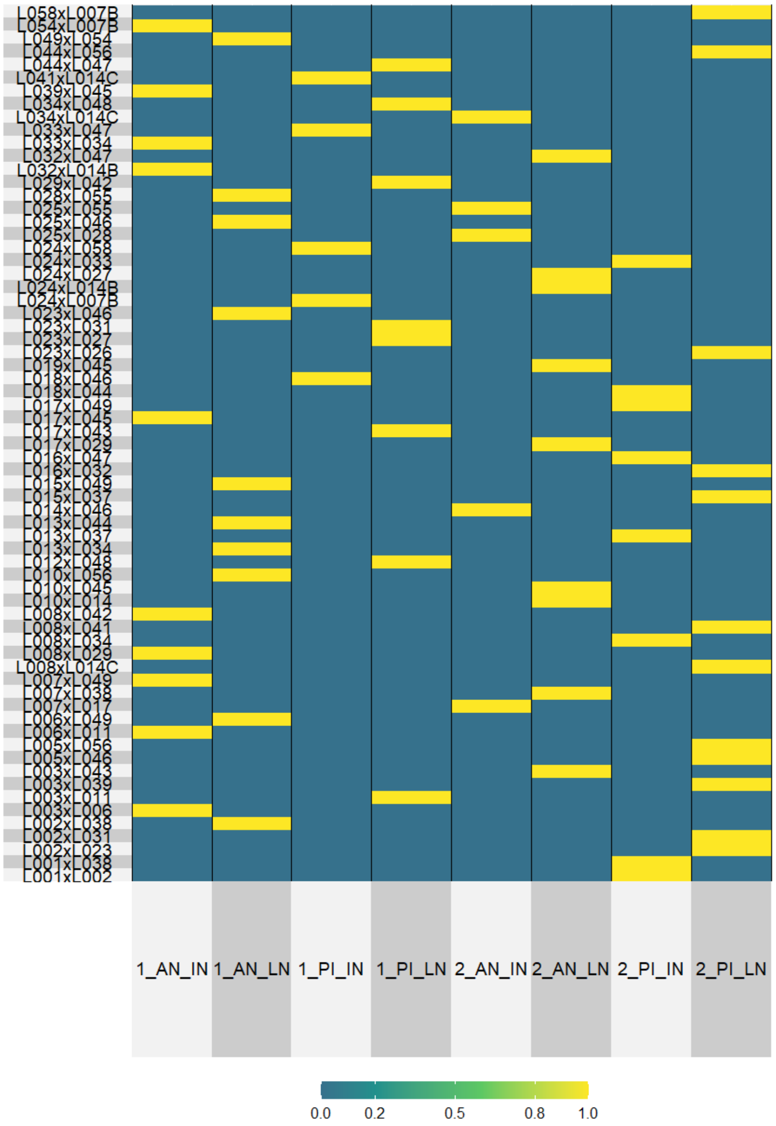

Supplementary Figure 3. Summary of the selective phenotyping approach drawn by the effective number of observations (N_{GE}) phenotyped in the field for the N-level Set (570 tropical maize hybrids over eight environments). The core of 67 maize hybrids (rows) per 8 environments (columns). In yellow is the hybrid-environment combinations phenotyped in the field-based trials. It resulted in N_{GE} = 67. At each environment, the number of genotypes were: 1_AN_IN (10), 1_AN_LN (10), 1_PI_IN (5), 1_PI_LN (8), 2_AN_IN (5), 2_AN_LN (9), 2_PI_IN (8), 2_PI_LN (12). The remaining 503 hybrids plus the blue cells were considered as testing set (virtual experimental network).
